# Identification of Epigenetically Altered Genes in Sporadic Amyotrophic Lateral Sclerosis

**DOI:** 10.1371/journal.pone.0052672

**Published:** 2012-12-26

**Authors:** Claudia Figueroa-Romero, Junguk Hur, Diane E. Bender, Colin E. Delaney, Michael D. Cataldo, Andrea L. Smith, Raymond Yung, Douglas M. Ruden, Brian C. Callaghan, Eva L. Feldman

**Affiliations:** 1 Department of Neurology, University of Michigan, Ann Arbor, Michigan, United States of America; 2 Department of Internal Medicine, University of Michigan, Ann Arbor, Michigan, United States of America; 3 Institute of Environmental Health Sciences, Eugene Applebaum College of Pharmacy and Health Sciences, Wayne State University, Detroit, Michigan, United States of America; 4 National Center for Integrative Biomedical Informatics, University of Michigan, Ann Arbor, Michigan, United States of America; Baylor College of Medicine, Jiao Tong University School of Medicine, United States of America

## Abstract

Amyotrophic lateral sclerosis (ALS) is a terminal disease involving the progressive degeneration of motor neurons within the motor cortex, brainstem and spinal cord. Most cases are sporadic (sALS) with unknown causes suggesting that the etiology of sALS may not be limited to the genotype of patients, but may be influenced by exposure to environmental factors. Alterations in epigenetic modifications are likely to play a role in disease onset and progression in ALS, as aberrant epigenetic patterns may be acquired throughout life. The aim of this study was to identify epigenetic marks associated with sALS. We hypothesize that epigenetic modifications may alter the expression of pathogenesis-related genes leading to the onset and progression of sALS. Using ELISA assays, we observed alterations in global methylation (5 mC) and hydroxymethylation (5 HmC) in postmortem sALS spinal cord but not in whole blood. Loci-specific differentially methylated and expressed genes in sALS spinal cord were identified by genome-wide 5mC and expression profiling using high-throughput microarrays. Concordant direction, hyper- or hypo-5mC with parallel changes in gene expression (under- or over-expression), was observed in 112 genes highly associated with biological functions related to immune and inflammation response. Furthermore, literature-based analysis identified potential associations among the epigenes. Integration of methylomics and transcriptomics data successfully revealed methylation changes in sALS spinal cord. This study represents an initial identification of epigenetic regulatory mechanisms in sALS which may improve our understanding of sALS pathogenesis for the identification of biomarkers and new therapeutic targets.

## Introduction

Amyotrophic lateral sclerosis (ALS) is a progressive and terminal neurodegenerative disease characterized by the selective degeneration of motor neurons within the motor cortex, brainstem and spinal cord [1]. In the United States, approximately 14 cases of ALS are diagnosed each day and 30,000 people are living with the disease. The average time from disease onset to death is 3 years and no treatment that substantially improves the clinical course of the disease is currently available [1].

Proposed pathogenic mechanisms of ALS include oxidative stress, glutamate excitotoxicity, impaired axonal transport, neurotrophic deprivation, neuroinflammation, apoptosis, altered protein turnover, and mitochondrial dysfunction [1], [2]. Moreover, influences from astrocytes and microglia in the motor neuron microenvironment contribute to pathogenesis [3]. In the last 20 years, a search for genetic factors has identified several genes associated with familial ALS (fALS) and a few with sporadic ALS (sALS) [4]–[6]. Because fALS only accounts for 5–10% of all cases of ALS, the causes leading to the vast majority of ALS (sALS) are poorly understood [1].

Environmental exposure to toxins, excessive physical activity, dietary factors, and changes in immunity increase the risk of developing sALS [7]. These factors may drive epigenetic changes, which are well suited to explain disease onset and progression in sALS, as they may be acquired throughout life. Epigenetic modifications, including covalent modifications of DNA and histones as well as RNA editing, dynamically regulate gene expression without altering the genetic code [8], [9]. These modifications are important in chromosome integrity, cellular differentiation, development, and aging [8], [10]. Two such modifications, 5-methylcytosine (5 mC) and 5-hydroxymethylcytosine (5 HmC) are associated with repression or activation of gene expression, respectively, in response to environmental and developmental factors linked to age-related diseases [11].

5mC at CpG (cytosine nucleotide separated by a phosphate from a guanine nucleotide) sites is a reversible mechanism facilitated by DNA (cytosine-5)-methyltransferases (DNMTs). Conversely, the Fe(II) and α-ketoglutarate (α-KG)-dependent ten-eleven translocation (TET) family of proteins catalyze oxidation and decarboxylation reactions of 5mC leading to 5-hydroxymethylcytosine (5 HmC), 5-formylcytosine (5 fC) and 5-carboxylcytosine (5 caC) [12], [13]. 5 HmC may be an intermediate for passive (during DNA replication) and active demethylation and/or serve as a docking site for proteins with high affinity for 5 HmC, thereby dissociating interactions between the transcriptional repression machinery and 5 mC [14].

In addition to the identification of alterations in global 5 HmC associated with sALS, this study represents one of the first methylation assessments of sALS by integrating methylome and transcriptome profiles of postmortem frozen human spinal cord samples. We identified differentially methylated sALS spinal cord genes exhibiting concordant mRNA expression overrepresented in functional categories implicated in sALS. These data support a role for epigenetic regulation in sALS and it may provide a better understanding of disease pathogenesis and facilitate the discovery of new therapeutic targets.

## Results

**Figure 1:**
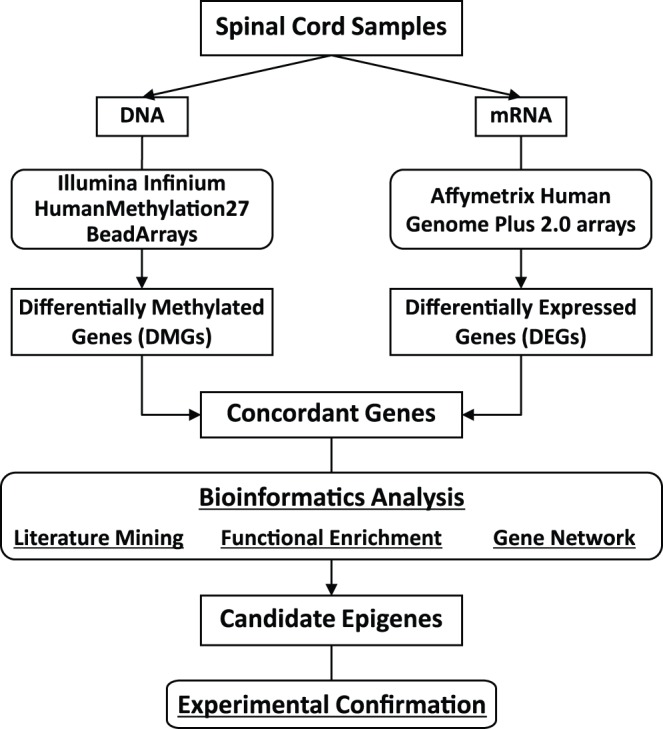
Flow chart for genome-wide epigenetic and expression analysis. DNA and RNA from postmortem human spinal cord samples were subjected to high-throughput epigenetic and gene expression screening, respectively. DMGs and DEGs representing concordant direction were further analyzed by bioinformatics analyses. Finally, identified candidate genes were experimentally confirmed in the spinal cord.

A workflow of our data analysis is provided in Fig. 1.

### Global 5mC is Increased in sALS Spinal Cord

**Figure 2:**
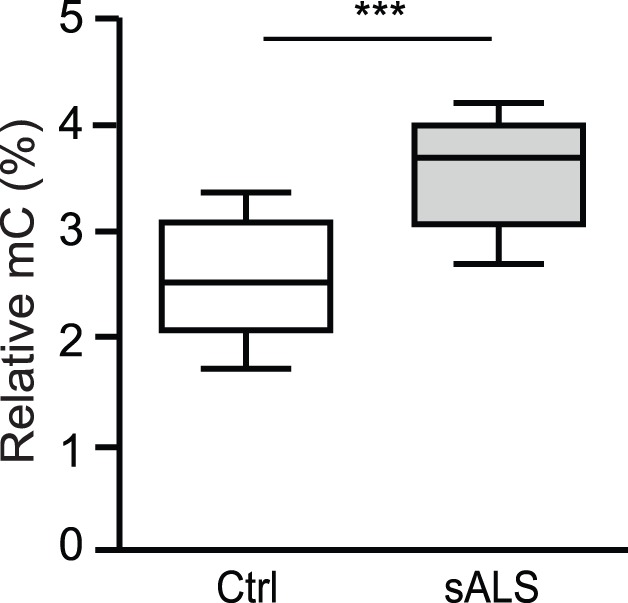
Global 5mC is increased in spinal cord of sALS. Genomic DNA extracted from control (open bar) or sALS (filled bar) postmortem human spinal cord was analyzed with an ELISA colorimetric assay for methylation, (5mC; Ctrl = 11, sALS = 11). Outliers, outside of median ±1.5 x Inter-Quartile-Range, were excluded. The data is presented as mean ± SEM of percent (%) 5mC using box and whiskers vertical bars plotting minimum to maximum values. A two-sample equal variance t-test used; ***p<0.001 compared to control group (Ctrl) with an achieved power of 99%.

Chestnut *et al.* recently reported an increase in DNMTs and 5mC immunoreactivity in ALS brain and spinal cord, suggesting that a global increase in 5mC is associated with the pathogenesis of ALS [15]. We assessed global 5mC of genomic DNA extracted from postmortem human spinal cord samples (sALS, n = 11; matching controls, n = 11; Tables 1, S1) using a colorimetric ELISA approach. We observed a modest but significant 1.4-fold increase in global 5mC in sALS (3.58±0.18) compared to controls (2.56±0.18) (*p = 0.0006*, Fig. 2), confirming previous observations [15].

**Table 1:** Demographics of the subjects for methylation and expression profiling of postmortem spinal cord.

Characteristics		sALS group	Control group	*p-value*
Number of subjects		12	11	–
Age (years)^(a)^		55 (35–71)	55 (36–73)	NS
Gender	Male	9	9	NS
	Female	3	2	–
Spinal cord location	Cervical	9	7	NS
	Thoracic	3	4	NS
Onset	Bulbar	1	0	–
	Limb	6	0	–
	NA	4	0	–
Postmortem interval (hours)^(b)^		14.5±7.0	15.2±6.2	NS
Cause of death	ALS	11	0	<0.001
	Accident	0	2	–
	PC	0	2	–
	ASCVD	0	5	–
	Cancer	0	1	–
	Cardiac arrest	0	1	–

(a) median (range); (b) mean ± standard deviation; PC, pulmonary complications; ASCVD, atherosclerotic cardiovascular disease; NS, not significant; NA, no data available.

### Genome-wide 5mC Profiling in sALS Spinal Cord

**Figure 3:**
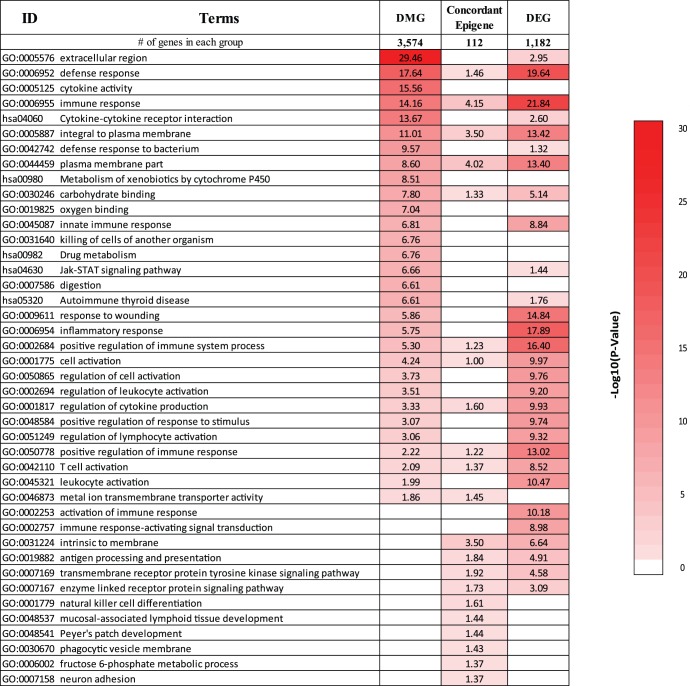
Biological functions of DMGs, DEGs, and concordant epigenes. Overrepresented biological functions were identified using DAVID. The top 20 biological functions ordered by p-value were collected from each gene set, and redundant terms were combined. The values in the table correspond to -log_10_ (DAVID p-value), ranging from 0 (white) to 30 (bright red). The values are not normalized across different gene sets with variable numbers of genes. The order of biological terms was based on the log-transformed p-values of DMGs and DEGs.

To identify loci-specific differentially methylated genes (DMGs) in sALS, postmortem spinal cord tissue (Tables 1, S1) was subjected to an unbiased genome-wide methylation screen using the Infinium Human Methylation27 DNA BeadChip (HM27K) array. We identified 4,261 significant differentially methylated autosomal CpG sites, representing 3,574 genes. Functional enrichment analysis identified biological categories of extracellular region *(p = 3.5E-30)*, defense response *(p = 2.3E-18)*, cytokine activity *(p = 2.7E-16)*, immune response *(p = 6.9E-15),* JAK-STAT signaling pathway *(p = 2.2E-7),* steroid hormone biosynthesis *(p = 3.6E-7),* and drug metabolism *(p = 3.1E-3)* were over-represented in spinal cord DMGs (Fig. 3). This suggests a role for epigenetic regulation in molecular mechanisms known to drive sALS pathogenesis [16]–[18]. Furthermore, the identified DMGs included previously reported epigenetically regulated genes such as runt-related transcription factor 3 (*RUNX3*), TNF-related apoptosis-inducing ligand (*TRAIL/TNFSF10*), *H19,* neuritin 1 (*NRN1)* and signal transducer and activator of transcription 5A (*STAT5A)* [19]–[23], validating our approach. The ALS-dependent differential methylation of three genes encoding the chemokines CKLF-like MARVEL transmembrane domain-containing proteins 2 and 3 (*CMTM2* and *CMTM3*) and the chemokine (C-X-C motif) ligand 12 (*CXCL12*), as well as the two transcription factors, *STAT5A* and the CCAAT/enhancer binding protein beta (*C/EBPB*) was assessed by pyrosequencing (Fig. S1). These results indicate parallel direction of differential methylation between the two assays.

### Genome-wide Expression Profiling in sALS Spinal Cord

An increase in promoter 5mC (hyper-methylation) is associated with gene silencing, while a decrease (hypo-methylation) reflects up-regulation of gene expression [8]. To identify functionally relevant epigenes, we performed genome-wide gene expression profiling of total RNA from sALS (n = 12) and control (n = 10) spinal cord (Tables 1, S1). We found 1,182 DEGs in sALS enriched with immune response-related biological categories such as defense response *(p = 2.3E-20)*, inflammatory response *(p = 1.3E-18)*, T-cell activation *(p = 3.0E-9*), leukocyte activation (*p = 1.6E-8*), cytokine binding *(p = 1.7E-7)* as well as carbohydrate binding *(p = 7.2E-6),* transmembrane receptor protein tyrosine kinase signaling *(p = 2.6E-5),* cell death *(p = 1.5E-3),* and JAK-STAT signaling *(p = 3.6E-2)* (Fig. 3). Several of these biological categories overlap with the 5mC profile, suggesting methylation may play a role in the regulation of these DEGs.

### Comparison between DMGs and DEGs in sALS Spinal Cord

**Figure 4:**
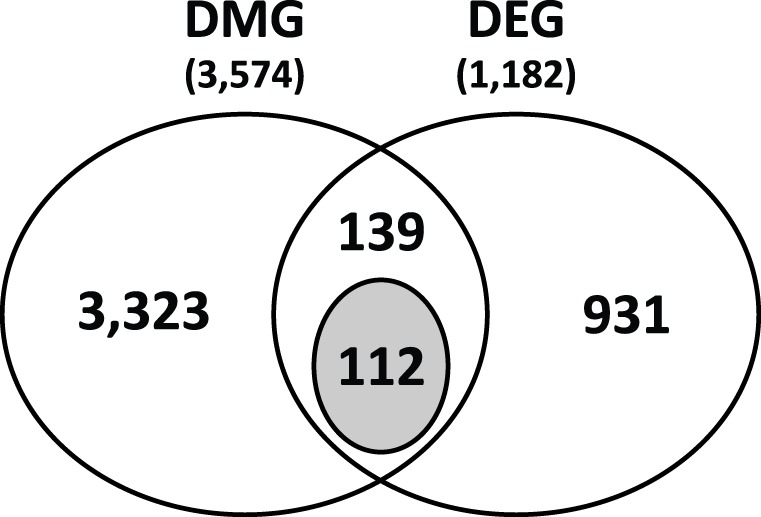
Overlapping of DMGs and DEGs. Identified DMGs and DEGs were compared for overlapping. Among 3,574 DMGs and 1,182 DEGs, 251 genes were common. Among these shared genes, 112 genes had concordant direction of change in methylation and expression (hyper-methylation with down-regulation or hypo-methylation with up-regulation) (shaded circle).

DMGs and DEGs were analyzed for their direction of change to select genes demonstrating concordant and potentially true regulation of gene expression by methylation. Between 3,574 DMGs and 1,182 DEGs, 251 genes were common (Table S2), of which approximately 70% were hyper-methylated as observed with global 5mC. Among these common genes, 112 epigenes demonstrated concordant direction of methylation and expression changes (hypo-methylation/up-regulation (51 genes; Table 2) or hyper-methylation/down-regulation (61 genes; Table 3) (Fig. 4). These concordant epigenes were enriched in biological categories including immune response *(p = 7.1E-5)*, plasma membrane part *(p = 9.6E-5)*, defense response *(p = 0.03)*, and neuron adhesion *(p = 0.05)* (Fig. 3).

**Table 2:** Hypo-methylated and up-regulated concordant epigenes.

GeneID	Description	Symbol	Δ5 mc (%)	DS	FC	SM	ALSoD& OS
58475	membrane-spanning 4-domains, subfamily A, member 7	MS4A7	6	−19.0	4.6		
1439	colony stimulating factor 2 receptor, beta, low-affinity	CSF2RB	6.6	−35.4	3.8		
9056	solute carrier family 7 (amino acid transporter light chain, y+L system), member 7	SLC7A7	6.6	−18.6	3.5		
822	capping protein (actin filament), gelsolin-like	CAPG	5.8	−13.9	3.4		
6556	solute carrier family 11 (proton-coupled divalent metal ion transporters), member 1	SLC11A1	8.6	−34.4	3.1		
54209	triggering receptor expressed on myeloid cells 2	TREM2	7.1	−26.0	2.9		
912	CD1d molecule	CD1D	5.6	−19.7	2.8		✓
10457	glycoprotein (transmembrane) nmb	GPNMB	10.1	−43.1	2.8	✓	
2203	fructose-1,6-bisphosphatase 1	FBP1	7.7	−96.5	2.7		
11006	leukocyte immunoglobulin-like receptor, subfamily B, member 4	LILRB4	4.6	−14.0	2.7		
27036	sialic acid binding Ig-like lectin 7	SIGLEC7	5.3	−15.9	2.7	✓	
4046	lymphocyte-specific protein 1	LSP1	6.4	−20.1	2.7		
864	runt-related transcription factor 3	RUNX3	7.5	−32.8	2.6		
11025	leukocyte immunoglobulin-like receptor, subfamily B, member 3	LILRB3	5.9	−14.0	2.5		
2207	Fc fragment of IgE, high affinity I, receptor for; gamma polypeptide	FCER1G	5.5	−20.2	2.4		
10261	immunoglobulin superfamily, member 6	IGSF6	10.2	−50.2	2.3		
286256	lipocalin 12	LCN12	5.2	−18.4	2.3		
1230	chemokine (C-C motif) receptor 1	CCR1	7.3	−25.2	2.3		
51225	ABI family, member 3	ABI3	6.3	-19.2	2.3		
1522	cathepsin Z	CTSZ	5.8	−39.3	2.2	✓	
26157	GTPase, IMAP family member 2	GIMAP2	7.2	−35.0	2.2		
10023	frequently rearranged in advanced T-cell lymphomas	FRAT1	5.2	−46.0	2.2		
1117	chitinase 3-like 2	CHI3L2	7.1	−22.6	2.1		
64344	hypoxia inducible factor 3, alpha subunit	HIF3A	5.8	−48.9	2.1		
10320	IKAROS family zinc finger 1 (Ikaros)	IKZF1	6.8	−22.2	2.1		
1051	CCAAT/enhancer binding protein (C/EBP), beta	C/EBPB	4.9	−54.7	2.1		
1601	disabled homolog 2, mitogen-responsive phosphoprotein (Drosophila)	DAB2	3.9	−13.7	2.0		
84898	plexin domain containing 2	PLXDC2	3	−23.7	2.0		
10990	leukocyte immunoglobulin-like receptor, subfamily B, member 5	LILRB5	7.5	−36.6	2.0		
254295	phytanoyl-CoA dioxygenase domain containing 1	PHYHD1	3.1	−13.6	1.9		
4488	msh homeobox 2	MSX2	5.4	−51.9	1.9		
558	AXL receptor tyrosine kinase	AXL	3.3	−14.5	1.8		
5359	phospholipid scramblase 1	PLSCR1	7.3	−82.6	1.8		
23526	histocompatibility (minor) HA-1	HMHA1	0	−33.5	1.8		
50856	C-type lectin domain family 4, member A	CLEC4A	9.5	−45.3	1.7		
116843	chromosome 6 open reading frame 192	C6orf192	3.4	−16.5	1.7		
55544	RNA binding motif protein 38	RBM38	4.9	−20.8	1.7		
23209	megalencephalic leukoencephalopathy with subcortical cysts 1	MLC1	7.4	−29.0	1.7		
23418	crumbs homolog 1 (Drosophila)	CRB1	6.2	−15.2	1.7		
1501	catenin (cadherin-associated protein), delta 2	CTNND2	2.9	−18.0	1.6		
5045	furin (paired basic amino acid cleaving enzyme)	FURIN	5.9	−19.7	1.6	✓	✓
1317	solute carrier family 31 (copper transporters), member 1	SLC31A1	5.3	−19.4	1.6	✓	
123920	CKLF-like MARVEL transmembrane domain containing 3	CMTM3	3.9	−16.6	1.6		
6776	signal transducer and activator of transcription 5A	STAT5A	4.2	−16.6	1.6		
57522	SLIT-ROBO Rho GTPase activating protein 1	SRGAP1	0.3	−43.9	1.5		
9404	leupaxin	LPXN	4	−13.9	1.5		
122618	phospholipase D family, member 4	PLD4	5.5	−18.0	1.5		
64859	oligonucleotide/oligosaccharide-binding fold containing 2A	OBFC2A	7.2	−99.8	1.5		
10628	thioredoxin interacting protein	TXNIP	4.3	−26.1	1.4		
79939	solute carrier family 35, member E1	SLC35E1	0	−46.6	1.3		
5660	prosaposin	PSAP	0	−33.5	1.3		

Genes are ordered by expression fold change (FC). Percent methylation change (Δ5 mc (%)) is calculated by |Beta_ALS_ – Beta_control_|/Beta_control_. DiffScore (DF), Differential methylation (a transformation of the p-value; p = 0.05, p = 0.01, p = 0.001 are equivalent to DiffScore = ±13, ±20, ±30, respectively). Fold change (FC) in gene expression ‘✓’ indicates that the corresponding gene was identified as an ALS-associated gene by SciMiner (SM), the ALSoD database, and other high-throughput microarray studies (OS) on human ALS spinal cord samples, respectively. See Table S2 more details.

**Table 3:** Hyper-methylated and down-regulated concordant epigenes.

GeneID	Description	Symbol	Δ5 mc (%)	DS	FC	SM	ALSoD& OS
7306	tyrosinase-related protein 1	TYRP1	9	50	−7.2	✓	
2706	gap junction protein, beta 2, 26 kDa	GJB2	16.3	18.4	−4.7		
26266	solute carrier family 13 (sodium/sulfate symporters), member 4	SLC13A4	4.6	26.2	−4.2		
4060	lumican	LUM	8.6	37	−4.1		✓
90523	muscular LMNA-interacting protein	C6orf142	6.7	343.9	−4.1		
149461	claudin 19	CLDN19	5.6	31.6	−4.1		
112464	protein kinase C, delta binding protein	PRKCDBP	6.3	28.6	−3.8		
195814	short chain dehydrogenase/reductase family 16C, member 5	SDR16C5	8.1	16.7	−3.5		
64073	chromosome 19 open reading frame 33	C19orf33	5.3	29.2	−3.4		
158038	leucine rich repeat and Ig domain containing 2	LINGO2	2.4	42.7	−3.3		✓
51299	neuritin 1	NRN1	3.9	18.1	−2.5	✓	
8727	catenin (cadherin-associated protein), alpha-like 1	CTNNAL1	6.4	17.1	−2.5		✓
51200	carboxypeptidase A4	CPA4	7.3	47.3	−2.4		
163782	KN motif and ankyrin repeat domains 4	KANK4	15.1	100.3	−2.4		
57664	pleckstrin homology domain containing, family A, member 4	PLEKHA4	11.9	26.4	−2.4		
283120	H19, imprinted maternally expressed transcript (non-protein coding)	H19	5.6	48.2	−2.3		
1755	deleted in malignant brain tumors 1	DMBT1	9.6	36.3	−2.3		
3045	hemoglobin, delta	HBD	7.8	77.9	−2.3		
8743	tumor necrosis factor (ligand) superfamily, member 10	TNFSF10	7.2	33	−2.2	✓	
4826	neuronatin	NNAT	5.8	21.1	−2.2		
2676	GDNF family receptor alpha 3	GFRA3	4.9	19.3	−2		
5121	Purkinje cell protein 4	PCP4	1.3	15.9	−2		✓
90139	tetraspanin 18	TSPAN18	5.2	24.8	−2		
5858	pregnancy-zone protein	PZP	9.5	48.7	−2		
10586	mab-21-like 2 (C. elegans)	MAB21L2	3.9	19.4	−1.9		✓
64288	zinc finger protein 323	ZNF323	8.6	32.6	−1.9		
84708	ligand of numb-protein X 1	LNX1	9.4	30.5	−1.9		
144347	family with sequence similarity 101, member A	FAM101A	6.9	33.9	−1.8		
25960	G protein-coupled receptor 124	GPR124	6.6	18.2	−1.8		
6335	sodium channel, voltage-gated, type IX, alpha subunit	SCN9A	6.5	47.3	−1.8	✓	
23089	paternally expressed 10	PEG10	10.1	21.2	−1.8		
254228	family with sequence similarity 26, member E	FAM26E	5.8	97	−1.8		
5332	phospholipase C, beta 4	PLCB4	6.7	49.5	−1.8		
168537	GTPase, IMAP family member 7	GIMAP7	7.8	31.1	−1.7		
4199	malic enzyme 1, NADP(+)-dependent, cytosolic	ME1	7.4	16.9	−1.7	✓	
135932	transmembrane protein 139	TMEM139	8.6	26.9	−1.7		
6932	transcription factor 7 (T-cell specific, HMG-box)	TCF7	5.3	87.4	−1.7		
79827	CXADR-like membrane protein	ASAM	9.7	87.6	−1.6		
152330	contactin 4	CNTN4	0.6	48.8	−1.6		✓
55228	PNMA-like 1	PNMAL1	8.8	30.6	−1.6		
150350	ENTH domain containing 1	ENTHD1	4.8	81.9	−1.5		
27237	Rho guanine nucleotide exchange factor (GEF) 16	ARHGEF16	7.5	101.6	−1.5		
57415	chromosome 3 open reading frame 14	C3orf14	7.9	78.2	−1.5		
84909	chromosome 9 open reading frame 3	C9orf3	0.1	40.4	−1.5	✓	
2326	flavin containing monooxygenase 1	FMO1	9.4	61.1	−1.5	✓	✓
5172	solute carrier family 26, member 4	SLC26A4	9.4	23.5	−1.5	✓	
9467	SH3-domain binding protein 5 (BTK-associated)	SH3BP5	7	50.6	−1.5		
166	amino-terminal enhancer of split	AES	5.8	42.7	−1.4		
3768	potassium inwardly-rectifying channel, subfamily J, member 12	KCNJ12	14.5	343.9	−1.4		
22927	hyaluronan binding protein 4	HABP4	12.2	45.1	−1.4		
8537	breast carcinoma amplified sequence 1	BCAS1	8.7	59.2	−1.4		
8817	fibroblast growth factor 18	FGF18	7.8	15.8	−1.4		✓
5213	phosphofructokinase, muscle	PFKM	5.9	29.3	−1.4		
85453	TSPY-like 5	TSPYL5	11.7	32	−1.4		
4233	met proto-oncogene (hepatocyte growth factor receptor)	MET	6	22.9	−1.4		
135250	retinoic acid early transcript 1E	RAET1E	9.6	89.7	−1.3		
2260	fibroblast growth factor receptor 1	FGFR1	5.1	18.7	−1.3	✓	
90135	BTB (POZ) domain containing 6	BTBD6	9.3	23.5	−1.3		
744	metallophosphoesterase domain containing 2	MPPED2	7.3	41.8	−1.3		
3749	potassium voltage-gated channel, Shaw-related subfamily, member 4	KCNC4	3.2	28.2	−1.3		✓
91977	myozenin 3	MYOZ3	5	15.3	−1.3		

Genes are ordered by expression fold change (FC). Percent methylation change (Δ5 mc (%)) is calculated by |Beta_ALS_ – Beta_control_|/Beta_control_. DiffScore (DF), Differential methylation (a transformation of the p-value; p = 0.05, p = 0.01, p = 0.001 are equivalent to DiffScore = ±13, ±20, ±30, respectively). Fold change (FC) in gene expression ‘✓’ indicates that the corresponding gene was identified as an ALS-associated gene by SciMiner (SM), the ALSoD database, and other high-throughput microarray studies (OS) on human ALS spinal cord samples, respectively. See Table S2 more details.

**Figure 5:**
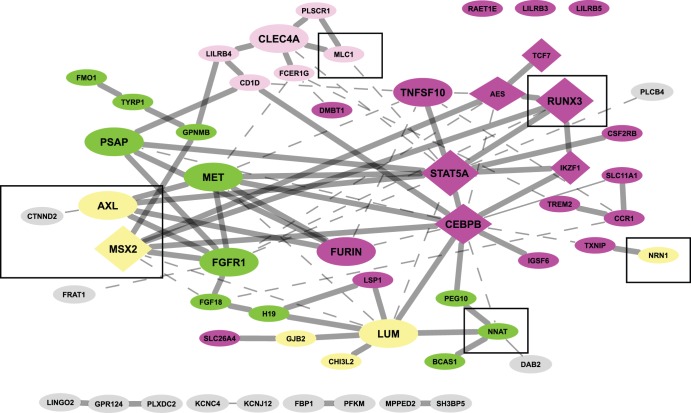
Literature-based association network of concordant epigenes. Literature-derived information for potential associations among the concordant epigenes was obtained using Genomatix Pathway System (GePS). Among the 112 concordant epigenes, 53 genes were co-cited at least once in PubMed abstracts at sentence-level (solid lines) or abstract-level (dashed lines). These epigenes were grouped by their representative biological role: immune response (dark pink), antigen presentation (light pink), tumor/suppressor related (green), extracellular matrix repair (yellow), and others (gray). Neuronal development-related genes are enclosed by a square. The epigenes with more than 5 connections to other genes are enlarged. A diamond-shape represents a transcription factor.

Gene co-citation network analysis revealed potential associations between some of the concordant genes identified in our screens. Among the 112 concordant epigenes, 53 genes were co-cited at least once in PubMed abstracts at the sentence or abstract-level. Within the co-citation network, the epigenes were classified into representative biological functions such as immune response, antigen presentation, tumor/suppressor related, and extracellular matrix repair (Fig. 5). In this network, the transcription factors *C/EBPB* and *STAT5A* were highly connected, suggesting a role in sALS pathogenesis. Moreover, some of these connections were directly or indirectly associated with concordant genes previously related to neuronal development such as muscle segment homeobox (msh) 2 (*MSX2*), megalencephalic leukoencephalopathy with subcortical cysts 1 (*MLC1*), *RUNX3*, catenin delta-2 (*CTNND2*), receptor tyrosine kinase (*AXL*), neuronatin *(NNAT),* and *NRN1* [24]–[30] (Fig. 5).

SciMiner identified 4,128 genes from ALS-related publications (as of 7/23/2012), which were compared to our 112 concordant epigenes. Fourteen genes were identified in two or more ALS-related publications with frequencies that were significantly different from those in over 20 million abstracts in PubMed *(p<0.05).* Fifty-one genes demonstrated ≥2-fold altered expression, including the chitinase 3-like protein 2 (*CHI3L2*), the triggering receptor expressed on myeloid cells-2 (*TREM2*), cathepsin Z (*CTSZ*), the lumican precursor protein (*LUM*), *H19*, and *TRAIL/TNFSF10* (Tables 2, 3). Thus, bioinformatics evaluation of the concordant epigenes identified by integrating methylomic and transcriptomic analyses detected both novel and previously known ALS-related genes.

### Experimental Confirmation using Real-time Polymerase Chain Reaction (RT-PCR)

**Figure 6:**
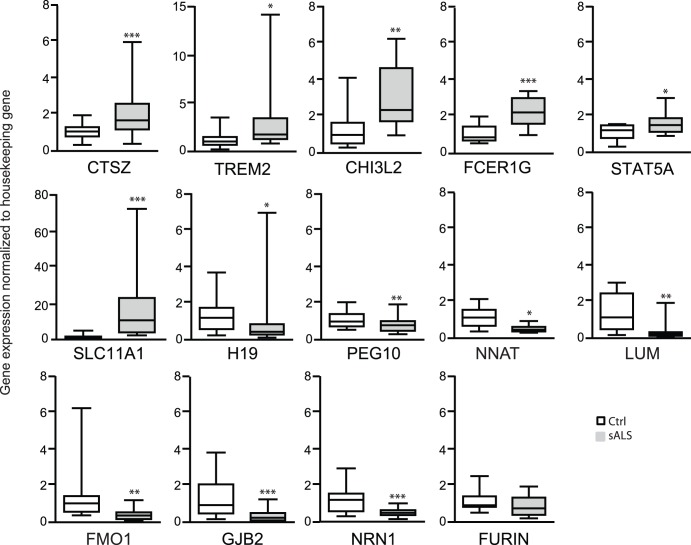
RT-PCR confirmation of concordant epigenes in spinal cord. RNA was extracted from the postmortem human spinal cord tissue that was used for the methylation analysis from sALS (n = 8–11) subjects and controls (n = 8–11) and subjected to RT-PCR. Results were normalized to glyceraldehyde-3- phosphate dehydrogenase (GAPDH) except for STAT5A which was normalized to TATA box binding protein (Tbp) and presented as fold changes calculated by the 2^−ΔΔC^_T_ method. Similar results were obtained when using different housekeeping genes (Fig. S2); *p<0.05, **p<0.01, ***p<0.001 compared to the control group (Ctrl). Data mean ± SEM is plotted using box and whiskers vertical bars plotting minimum to maximum values.

Expression of 14 concordant epigenes selected either from the ALS-related literature or from the expression array data was confirmed by RT-PCR (Figs. 6 and S2, Table 4). Of the genes previously related to the ALS literature, *NRN1*, *FMO1,* and the lumican precursor protein *(LUM)* were under-expressed, while the lysosomal protease *CTSZ* was over-expressed in sALS. No significant difference between sALS and control subjects was observed for FES-upstream region (*FURIN)*. Novel sALS-associated epigenes such as *STAT5A, TREM2*, the high-affinity IgE receptor (*FCER1G*), *CHI3L2*, and the proton-couple divalent metal ion transporter solute carrier family 11 member 1A (*SLC11A1*) were over-expressed in sALS. *SLC11A1* presented the highest increase by 12.7-fold. Down-regulation was validated for gap junction ß-2 (*GJB2*)/Connexin-26 as well as imprinted genes such as *H19, NNAT,* and the paternally expressed 10 (*PEG10*). In summary, the RT-PCR expression data indicate high concordance with the microarray expression data, validating our results.

**Table 4:** Confirmation of microarray differential expression in spinal cord using RT-PCR.

Symbol	Control	sALS	*<p-value*	Fold-Change	Microarray Fold-Change	Confirmed
*CTSZ*	1.10±0.07	2.06±0.23	*0.001*	1.9	2.2	Yes
*STAT5A*	1.10±0.12	1.58±0.20	*0.05*	1.4	1.6	Yes
*TREM2*	1.20±0.17	3.18±0.72	*0.05*	2.6	2.9	Yes
*FCER1G*	1.10±0.11	2.30±0.19	*0.001*	2.1	2.4	Yes
*CHI3L2*	1.32±0.29	3.13±0.48	*0.01*	1.6	2.1	Yes
*SLC11A1*	1.34 0.23	17.00±3.38	*0.001*	12.7	3.1	Yes
*NRN1*	1.19±0.18	0.49±0.05	*0.001*	−2.4	−2.5	Yes
*NNAT*	1.14±0.20	0.52±0.06	*0.05*	−2.2	−2.2	Yes
*FMO1*	1.41±0.40	0.38±0.07	*0.001*	−3.7	−1.5	Yes
*GJB2*	1.38±0.27	0.35±0.07	*0.001*	−3.9	−4.7	Yes
*FURIN*	1.11±0.16	0.83±0.13	*0.17*	−1.3	1.6	No
*H19*	1.28±0.15	0.68±0.20	*0.05*	−1.9	−2.3	Yes
*PEG10*	1.08±0.08	0.79±0.07	*0.01*	−1.4	−1.8	Yes
*LUM*	1.40±0.29	0.32±0.13	*0.01*	−4.4	−4.1	Yes

‘Yes’ indicates the differential expression of the corresponding gene is statistically significant and demonstrated the same direction of change as in the microarray data.

### Global 5 HmC Increases in sALS Spinal Cord

**Figure 7:**
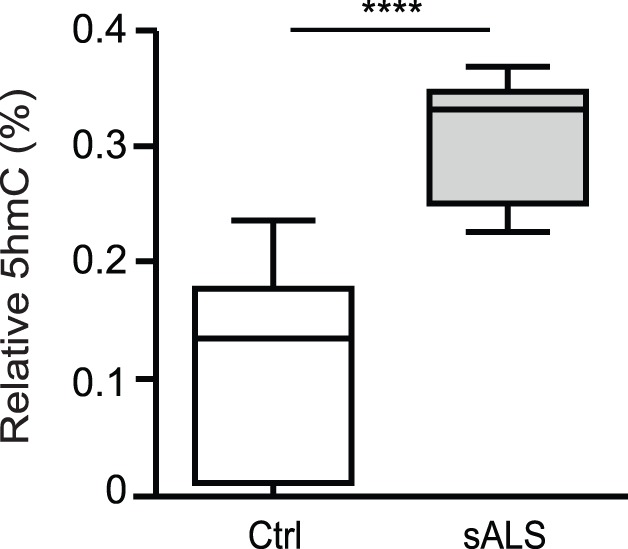
Global 5 HmC is increased in spinal cord of sALS. Genomic DNA extracted from control or sALS postmortem human spinal cord was analyzed with an ELISA colorimetric assay for hydroxymethylation (5 HmC; Ctrl = 9, sALS = 8). The data is presented as mean ± SEM of percent (%)5 HmC using a two-sample equal variance t-test and plotted using box and whiskers vertical bars with minimum to maximum values; ****p<0.0001 compared to the control group (Ctrl).

5 HmC, an alternate epigenetic modification of DNA, is increased in brain compared to other human tissues, and alterations in global 5 HmC are associated with age-related neurodegenerative disorders, suggesting an important role of 5 HmC in neuronal the function [31]–[34]. We measured global 5 HmC for sALS and control spinal cord samples previously analyzed for global 5mC. We observed an approximately 3.0-fold increase in global 5 HmC in sALS (0.31±0.02) compared to controls (0.11±0.03) (*p<0.0001*) (Fig. 7). This is the first report of aberrant levels of global 5 HmC associated with the pathogenesis of sALS.

### Global 5mC and 5 HmC in sALS Whole Blood

**Figure 8:**
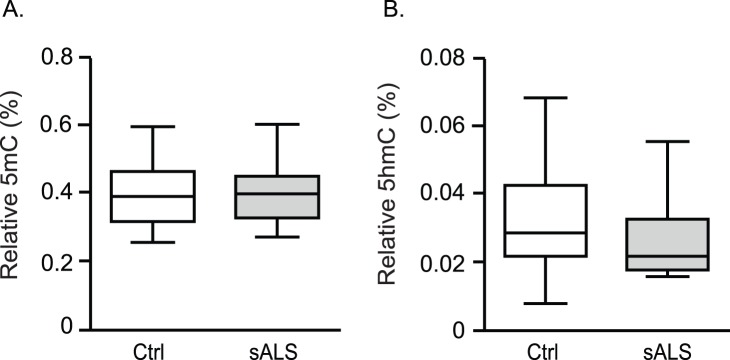
Changes in global 5 HmC and 5mC are not detected in ALS whole blood. Genomic DNA extracted from control or sALS human whole blood was analyzed for 5mC (Ctrl n = 11, ALS n = 11*; p = 0.94*) and 5 HmC (Ctrl n = 11, sALS n = 11; *p = 0.40*). Percent (%) 5mC and 5 HmC is presented as mean ± SEM using a two-sample equal variance t-test and graphed using box and whiskers vertical bars plotting minimum to maximum values.

High correlation of epigenetic marks in spinal cord and blood may be useful for diagnostic and therapeutic application in ALS. We investigated whether global 5mC and 5 HmC would be altered in sALS whole blood similarly to spinal cord. Whole blood genomic DNA from a different cohort (Tables 5, S1) was subjected to global 5mC and global 5 HmC by ELISA. The levels of percent global 5mC and 5 HmC in whole blood were 10 fold lower compared to spinal cord, in agreement with recent reports [33], [35]. Contrary to spinal cord, no differential percent global 5mC (controls, 0.408±0.300; ALS, 0.405±0.027 *p = 0.941*) and global 5 HmC (controls, 0.033±0.005; ALS, 0.032±0.004; *p = 0.401*) (Fig. 8) were observed in whole blood.

**Table 5:** Characteristics of sALS and control subjects used for global 5mC and 5 HmC in whole blood.

Characteristics		sALS group	Control group	*p-value*
Number of subjects		11	12	–
Age (years)(a)		60 (48–68)	59 (48–70)	NS
Gender	Male	7	7	NS
	Female	4	5	NS
Onset	Bulbar	3	0	–
	Limb	8	0	–
Disease duration (months)^(a)(b)^		45 (16–64)	–	–
Health condition of controls(c)	Anxiety	0	1	–
	Asthma	0	2	–
	Cancer	0	3	–
	Healthy	0	5	–
	Hearing loss	0	1	–
	Hypercholesterolemia	0	1	–
	Hypothyroidism	0	2	–
	Hypertension	0	1	–

(a) median (range);

(b) mean ± standard deviation;

(c) some patients presented more than one condition; NS, not significant; ‘–‘, value not available.

## Discussion

Although several genes have been implicated in the pathogenesis of ALS, the causes leading to most cases remain unknown. Environmental factors may be associated with the onset and development of sALS by altering epigenetic regulation [7], [8]. The aim of this study was to identify sALS-associated epigenetic marks resulting in aberrant gene expression. Abnormal 5mC patterns of repetitive elements such as Alu and LINE1, as well as altered function of methylation regulators such as the DNMTs, lead to changes in global 5mC or 5 HmC associated with neurodegeneration [15], [36]. We demonstrate increased global methylation in sALS spinal cord, perhaps due to an increase in DNMT activity [15]. Furthermore, we report for the first time an increase in global 5 HmC in sALS spinal cord. Increased 5mC and 5 HmC may be due to 5mC providing more substrate for the TET proteins [10], TETs are not differentially expressed in spinal cord sALS according to our microarray data (data not shown). TET should decrease the amount of 5mC only if 5mC is not increasing at a faster rate than the oxidation reaction. Although normal aging leads to increased global 5 HmC in mouse hippocampal DNA independently of increased levels of oxidative stress [31], in ALS, increased oxidative DNA damage and free radicals may contribute to global 5 HmC dysregulation. The base excision repair (BER) pathway responsible for oxidative DNA damage restoration and one of the active demethylation pathways, is deficient in ALS [14], [37], [38].

Methylomics and transcriptomics analyses identified potential biologically relevant epigenes in postmortem sALS spinal cord. These epigenes were enriched with biological functions related to inflammation and the immune responses, previously linked to ALS [39]–[41]. Our data suggest that alterations in gene expression of immune-related genes in sALS may be regulated by methylation. Immune-related concordant epigenes including *TREM2*, chemokine (C-C motif) receptor 1/RANTES receptor (*CCR1*), *SLC11A1*, the transmembrane receptor C-type lectin domain family 4 member A isoform 1 (*CLEC4A*), and the IgE receptor (*FCER1G*) were found to be over-expressed in sALS. Our findings suggest an infiltration of myeloid cells, mast cells, or natural killer cells to the damaged area and/or activation of resident microglia [42]–[44]. Supporting our observations, neuro-inflammation was recently associated with systemic macrophage activation independent of T-cell activation and the recruitment of activated inflammatory monocytes to the spinal cord in ALS [45], [46]. Although immunosuppressive and anti-inflammatory therapies have shown to delay disease onset in ALS animal models, clinical trials have not revealed a major effect on disease progression or survival [46]–[50]. This suggests that continuous activation of microglia leading to neuronal damage surpasses the capacity of the nervous system to respond to immunosuppressive and anti-inflammatory therapies at later stages of ALS, implicating a need for biomarkers identifying early immune-related changes in sALS.

Co-citation network and literature mining approaches identified connections among novel and previously implicated ALS-related epigenes and pathways [51], [52]. The transcription factors *STAT5A* and *C/EBPB* are highly connected in our co-citation network and their interplay promote activation of various genes including interleukin-6 (*IL-6*) [53]. Moreover, recent reports implicate *C/EBPB* and *STAT5A* in ALS pathogenesis and neurodegeneration. For instance, expression of *C/EBPB* in ALS microglia from spinal cord suggests an important role of C/EBPB in the regulation of neurotoxic genes in the ALS neuronal microenvironment [45], [54]. Furthermore, changes in *STAT5A* expression may reflect an altered inflammatory response contributing to the pathogenesis of ALS. Over-expression of *STAT5A* reduces neuronal degeneration associated with spinal muscular atrophy, a neurodegenerative disease with similar pathogenesis as ALS, and it provides oligodendrocyte protection, which in turn favors neuronal environment preservation [55]–[57]. Whether positive regulation of *STAT5A* in sALS is due to an anti-apoptotic response to compensate for the degeneration of the nervous system, or its over-expression is responsible, in part, for the pathogenesis of the disease remains to be determined. Interestingly, we observed potential transcription factor binding sites (TFBSs) for STAT5A and C/EBPB in 40% and 48% of the promoters of our identified DEGs, respectively; the binding sites for STAT5A and C/EBPB are 1.2 (*p = 4.1E-12*) and 1.3 *(p = 3.8E-13)* times more frequent in the DEGs than in the vertebrate promoters, respectively. Our observations suggest epigenetic mechanisms, in part, drive the expression of central regulators of downstream targets in sALS.

Our study identified ALS-dependent methylation dysregulation of several genes previously implicated in neuronal development, differentiation, and proliferation, including Slit-Robo Rho GTPase activating protein 1 (*SRGAP1)*, crumbs homolog 1 (*CRB1*), *MSX2*, *MLC1*, *CTNND2*, *AXL*, *RUNX3*, *NNAT*, and *NRN1* [24]–[30], [58], [59]. Altered expression and/or mutations in *CRB1*, *MLC1*, and *CTNND2* are associated with mental retardation and neurodegeneration [26], [28], [60], [61]. Moreover, we found *CRB1* to be hypo-methylated and up-regulated in sALS, in agreement with overexpression observed by Kudo *et al.* in spinal cord from a mouse model of familial ALS (*SOD1* G93A) as well as human sALS [61]. Interestingly, most of these genes were identified by our literature-based association network of concordant epigenes and were connected to *C/EBPB* and *STAT5A*. Analysis of the promoter region of these genes indicates a high incidence of potential TFBSs for these two transcription factors, suggesting a potential role of *STAT5A* and *C/EBPB* in the regulation of neuronal genes in the pathogenesis of sALS. Our observations suggest sALS-related alterations in methylation may lead to aberrant expression of genes required for neuronal homeostasis. Nevertheless, more studies need to be done to address the role of methylation, *STAT5A*, and *C/EBPB* in the regulation of neuronal genes.

Another sALS-related epigene, *CTSZ* warrants further investigation since its aberrant expression is associated with neurodegeneration by promoting neurotoxin elimination in the damaged cellular environment [62]. Expression of *CTSZ* as well as two other members of the cathepsin family, cathepsins B and D, increases in human and rodent ALS spinal cord and mutant *SOD1* (G86R, G93A) mouse skeletal muscle suggesting they play an important role in ALS [63], [64].

Except for optineurin (OPTN) [65], which was identified as a hypo-methylated DMG without demonstrating changes in gene expression, loci known to be mutated in fALS were not present in our concordant epigenes [4]. This agrees with recent studies indicating that promoter regions of *SOD1*, *VEGF*, and metallothioneins I and II are not differentially methylated in sALS [66], [67]. When compared with the ALS Online genetics Database (ALSoD)-reported genes and other ALS-dependent methylation/gene expression of profiling studies [39], [41], [68]–[72], we observed a modest overlap of four concordant epigenes; Purkinje cell protein 4 (*PCP4)*, catenin (*CTNNAL1*), fibroblast growth factor 18 (*FGF18*), and flavin containing monooxygenase 1 (*FMO1*). Furthermore, five of our concordant genes presented opposite direction of expression when compared to known ALS-dependent differentially expressed gene. Our data indicate that epigenetic mechanisms are potential regulators of these key genes in ALS.

Based on the large number of genes identified in the methylation (3,574 genes) and expression (1,182 genes) arrays, relatively few sALS-associated genes presented concordant direction between methylation and gene expression. The low occurrence of a small subset of genes potentially regulated by CpG modification in such a way that hyper-methylation promotes gene silencing and hypo-methylation promotes gene expression has been previously documented [73]. 5mC within promoter regions is associated with repression of gene expression by interfering with transcription factor binding or by providing a binding site for transcriptional repressors [10]. Interestingly, over half (55%) of the 251 common DMGs/DEGs presented same direction of 5mC and expression. In some cases, 5mC positively regulates gene transcription by promoting transcription factor binding at promoter regions [74] or, more commonly, by modifying intragenic CpG sites facilitating transcription efficiency, histone conformation, and regulating levels of sense and antisense mRNA [75]. Furthermore, 5 HmC, a highly enriched modification in brain, correlates with increased gene expression [10]. HM27K does not differentiate between 5mC and 5 HmC; therefore, some of the common epigenes presenting same direction of methylation and expression may be regulated by 5 HmC.

Although the high incidence of same direction sALS concordant epigenes parallels the high levels of global 5 HmC in spinal cord, loci specific 5 HmC modifications associated with sALS remain to be identified. Gene expression of non-common DEG (non-DMGs) could be determined, in part, by 5mC-dependent regulation of transcription factors. In addition to *STAT5* and *C/EBPB*, we identified several transcription factors as concordant genes such as the transcription factor 7 (*TCF7*), *RUNX3*, IKAROS family zinc finger 1 (*IKZF1*), *MSX2*, and hypoxia inducible factor 3, alpha subunit (*HIF3A*). Furthermore, regulation of gene expression is a dynamic and complex mechanism and the interplay of several epigenetic pathways has been reported to modulate adult neurogenesis [76]. Therefore, alterations to epigenetic networks in conjunction with genetic predisposition may result in the development of sALS.

The prospect of identifying sALS epigenetic biomarkers in blood is exciting as it provides a minimally invasive alternative for sALS diagnostic and prognostic assessments. Although we did not detect significant global 5mC and 5 HmC differences in blood and inflammation-related epigene biomarkers may reflect systemic inflammatory changes rather than neuronal changes, further investigation of individual loci may provide potential epigenetic biomarkers for sALS.

There were several limitations to our study. First, a relatively small number of samples were analyzed and loci-specific 5 HmC analysis is still needed. Nevertheless, this is an initial step towards identifying epigenetic mechanisms altering key pathways leading to sALS, which will be validated in larger cohorts. Second, sALS postmortem tissue reflects the terminal disease stage rather than the pathogenic mechanisms leading to disease onset and progression. As sALS-affected motor neurons deteriorate at the terminal stage and heterogeneous tissue consisting of both gray and white matter was analyzed, our results may represent epigenetic regulation of the neuronal microenvironment, including microglia activation and the scarce neurons surviving the degenerative process [54], [72]. This may explain, in part the discrepancy in the direction of expression of common and concordant genes reported here with other sALS genome-wide expression profiles, as well as the heavily represented inflammation-related genes, in our concordant epigenes, which are not differentially expressed specifically in sALS motor neurons or ventral horns [68]. Finally, more studies are needed to concretely identify whether or not the genes identified in this study are involved in ALS pathogenesis.

Advances in identifying epigenetic regulators in disease states have led to new therapeutic approaches. Interestingly, demethylating agents have been extensively studied to reverse aberrant epigenetic changes associated with cancer [77] and more recently, histone deacetylase inhibitors have shown to have neuroprotective properties in animal models of neurodegenerative diseases [78]. These observations suggest reversible epigenetic modifications carry the potential for therapeutic treatment in sALS. We contend that environmental life exposures result in failure to maintain epigenetic homeostasis in the nervous system microenvironment leading to global and loci specific aberrant regulation of gene expression in sALS-affected tissue. Ascertaining the role of epigenetic regulation may provide a better understanding of the pathogenesis of sALS and new therapeutic targets.

## Methods

### Subjects and Tissue

Frozen human spinal cord samples from 12 Caucasian sALS subjects and 11 age and gender-matched neurologically-normal controls were obtained from the National Center for Child Health and Human Development (NICHD) Brain and Tissue Bank for Developmental Disorders at the University of Maryland, Baltimore, MD (Table 1). Whole blood was collected in EDTA tubes from a different cohort of 11 Caucasian sALS and 11 age- (5±years) and gender-matched neurologically-normal control subjects at the University of Michigan ALS Consortium (Table 4). Table S1 summarizes the samples used for each assay.

### Ethics Statement

The participants donating blood reviewed and signed a written informed consent under a protocol reviewed and approved by the University of Michigan Medical School Institutional Review Board (HUM00028826).

### Nucleotide Extraction and DNA Bisulfite Conversion

Genomic DNA was extracted from 50 mg of frozen postmortem spinal cord tissue (mostly grey matter including the ventral horn, but some white matter included) using the Promega Maxwell 16 Tissue DNA Purification kit and a Maxwell instrument (Promega Co, Madison, WI). Genomic DNA (1 µg) was bisulfite converted with an EZ DNA Methylation Kit (Zymo Research, Irvine, CA) according to the manufacturer’s instructions. Total RNA was extracted from the same tissue for methylation profiling (Table S1) using the RNeasy kit and treated with RNAse-free DNase1 according to the manufacturer’s instructions (Qiagen, Valencia, CA). Automated genomic DNA extraction from whole blood was performed at the Michigan Institute for Clinical & Health Research (MICHR) at the University of Michigan using Autogen FlexStar (Autogen, Holliston, MA) and Qiagen Flexigene reagents. Nucleotide concentration was assessed using a Nanodrop 2000 (Thermo Scientific) and RNA integrity was determined by microfluid electrophoresis with a 21000 Bioanalyzer (Agilent Technologies, Palo Alto, CA).

### Global 5mC and 5 HmC

Differences in genomic DNA global methylation (Global 5mC) and hydroxymethylation (Global 5 HmC) from sALS and control spinal cord or whole blood were determined in duplicate using the colorimetric enzyme-linked immuno-sorbent assay (ELISA) MethylFlash (Methylated or Hydroxymethylated) DNA Quantification Kits according to the manufacturer’s directions (Epigentek Group Inc., Farmingdale, NY). The absorbance at 450 nm was captured in a Fluoroskan Ascent microplate reader (Labsystems, Vienna, Va). The percentage of Global 5mC and 5 HmC is expressed as mean ± standard error mean (SEM). The two-tailed t-test was used for statistical comparison. Graphs and statistical analysis were obtained with GraphPad Prism 5.

### Methylation Profiling and Identification of DMGs

For high-throughput methylation profiling, 200 ng of bisulfite-converted DNA was whole-genome amplified (WGA), enzymatically fragmented, purified, and hybridized to the Infinium Human Methylation27 DNA BeadChip array (HM27K; Illumina, Inc., San Diego, CA) following the manufacturer’s instructions at the University of Michigan Sequencing Core. The HM27K quantitatively determines DNA methylation for 27,578 CpG sites spanning 14,495 genes. DMGs were identified using Illumina’s GenomeStudio software [79]. Single-base resolution corresponding to DNA methylation levels for each locus was reported and the methylation level is given by a beta (ß) value describing the percentage of the degree of methylation ranging from 0 (no methylation) to 1 (complete methylation). Any methylation value with a detection P-value >0.05 was excluded. Differential methylation of the selected CpG target regions of autosomal chromosomes between sALS and control groups were tested using Illumina Custom algorithm with multiple testing corrections applied. DiffScore, GenomeStudio’s statistical significance score for differential methylation, of >13 for hyper-methylation or <-13 for hypo-methylation, equivalent to False Discovery Rate (FDR) <5%, were used.

### Genome-wide Expression Profiling

Microarray gene expression analysis was performed as previously described in our published protocols [80]. Briefly, RNA samples with an RNA integrity number (RIN) >6.4 were used for further microarray and real-time PCR analysis. Total RNA (75 ng) was amplified and biotin-labeled using the Ovation Biotin-RNA Amplification and Labeling System (NuGEN Technologies, Inc., San Carlos, CA) according to the manufacturer’s instructions. Amplification and hybridization was performed at the University of Michigan DNA Sequencing Core Affymetrix and Microarray Core Group (Ann Arbor, MI) using the Affymetrix GeneChip Human Genome U133 Plus 2.0 Array measuring over 47,000 transcripts representing over 20,000 human genes.

Affymetrix CEL files were analyzed using a local version of the GenePattern genomic analysis platform from the Broad Institute [81]. Samples were Robust Multi-array Average (RMA) normalized using the BrainArray Custom CDF HGU133Plus2_Hs_ENTREZG version 14 [82]. Microarray quality was assessed as previously published [80]. Briefly, probe-level modeling (PLM) and quality metrics provided by the BioConductor affy package were used to identify low-quality arrays [83]–[85]. Outlier arrays, skewed away from other arrays, identified by Principal Component Analysis (PCA) were excluded from further analyses. Intensity-Based Moderated T-statistic (IBMT) [86] was employed to identify DMGs with a 10% FDR cut-off between sALS and control samples.

### Identification of Differentially Expressed Genes (DEGs)

Concordant epigenes are those exhibiting significant differential methylation (hyper- or hypo-methylation) and a parallel change of gene expression (under- or over-expression, respectively) between sALS and control. Differentially methylated (DMGs)/expressed (DEGs) genes were subjected to bioinformatics analyses.

### Bioinformatics Analysis of Concordant Epigenes

#### Functional enrichment analysis

Database for Annotation, Visualization and Integrated Discovery (DAVID; http://david.abcc.ncifcrf.gov/) [87], [88] was used to identify enriched molecular biological functions and ALS-relevant pathways of concordant epigenes. A Benjamini-Hochberg corrected P-value of 0.05 was used as the cut-off for statistically significant over-representation.

#### Literature mining analysis

A literature mining approach was used to obtain a comprehensive list of potential ALS-associated targets (genes/proteins). SciMiner, a web-based literature mining tool [89], [90], retrieves, processes documents, and identifies potential ALS-associated targets from the ALS-related literature, defined by a PubMed-style query of “Amyotrophic Lateral Sclerosis”. The concordant epigenes were compared against the literature-derived ALS-associated targets that were observed in at least 2 or more papers and whose frequency (in terms of the number of papers) was significantly different from the background. Fisher’s exact test *(p-value <0.05)* was used to determine whether each gene’s frequency was significantly different from the complete collection of abstracts of over 20 million papers in PubMed. The concordant genes identified by the high-throughput arrays were compared with these literature-derived ALS-related genes to identify which known disease-relevant genes are most highly methylated/expressed and, consequently, likely involved in disease pathogenesis. The resulting genes were designated as literature-derived ALS-associated epigenes.

#### Transcriptional network analysis

To elucidate the functional relationships among the concordant epigenes, we generated transcriptional networks using Genomatix Pathway Systems (GePS; Genomatix Software GmbH, Munich, Germany) with a sentence-level co-citation filter. Two genes co-cited at the sentence level in the literature are linked, resulting in a co-citation network. Additionally, transcriptional regulatory information of predicted transcription factor binding sites (TFBS) in promoter regions of genes could be further incorporated. The network allows the visualization of concordant epigenes, their potential associations, and transcriptional regulation with each other. Therefore, it helps in the identification of key genes that are highly connected to genes, and which play potentially important roles in the pathogenesis of sALS. Potential TFBSs of two highly connected genes in the network, *STAT5A* and *C/EBPB,* were searched among the promoters of the concordant epigenes using MatInspector (Genomatix) [91].

### Pyrosequencing

To validate the HM27K arrays, we assessed gene-specific methylation of three selected cytokine genes based on the fact that immune response is associated with the pathogenesis of sALS [92] and two transcription factors. Amplicons of the promoter regions of the genes coding for the CKLF-like MARVEL transmembrane domain-containing proteins 2 and 3 (CMTM2 and CMTM3), the chemokine (C-X-C motif) ligand 12 (CXCL12), signal transducer and activator of transcription 5A (STAT5A), and CCAAT/enhancer binding protein beta (C/EBPB) were generated in 30 ml reactions using the PyroMark kit (Qiagen, Valencia, CA) with 4.8 pmol of the forward non-biotinylated primer, 2.4 pmol of the reverse biotinylated primer (Table S3), and 25 ng of bisulfite converted genomic DNA as previously described [93]. PCR conditions: 95°C for 15 min, 50 cycles [95°C for 30 s, 40–50°C for 30 s, 72°C for 20 s], 72°C for 10 min. Ten ml of the amplicon was Streptavidin Sepharose (Amersham Bioscience, Uppsala, Sweden) were purified, denatured with 0.2 M NaOH, and pyrosequenced using 0.5 mM of sequencing primer in a PSQ96 HS System (Qiagen) following the manufacturer’s protocol. Percent methylation of the region analyzed containing the identified Illumina methylation site or individual sites are presented as mean ± SEM with a two-sample equal variance t-test using GraphPad Prism 5.

### RT-PCR

cDNA was generated by reverse transcription from total RNA isolated for microarray analysis using an iScript cDNA Synthesis Kit (Bio-Rad, Hercules, CA). RT-PCR was performed in triplicate using sequence-specific primers (Table S4) with SYBR Green PCR reagents (Bio-Rad, Hercules, CA). The PCR amplification profile was as follows: 95°C for 5 min, [denaturation at 95°C for 30 s, annealing at 55–60°C for 60 s, and extension at 72°C for 30 s] x40 cycles, and a final phase of 72°C for 5 min. The fluorescence threshold C_T_ value, representing mRNA expressed in sALS samples, was calculated by the iCycler iQ system software. mRNA levels were normalized to an endogenous reference (ΔC_T_) and then relative to the control group (ΔΔC_T_). Levels of PCR products are demonstrated as mean ± SEM and a two-sample equal variance t-test was performed using GraphPad Prism 5 to confirm that mRNA levels were significantly different between sALS and control.

## Supporting Information

Figure S1 **Validation of HM27K arrays using pyrosequencing.** Amplicons to the promoter regions identified by HM27K of cytokines *CXCL12*, *CMTM3*, C*MTM2, C/EBPB*, and *STAT5A* were generated using bisulfite-converted genomic DNA from human postmortem spinal cord and used as templates for pyrosequencing (ALS n = 11; Ctrl n = 11) Results are presented as mean of percent methylation of all CpG sites within the area tested on each gene (A) or as percent methylation of individual sites for *STAT5A*; site 2 was identified with the HM27K (B). Results are presented as mean ± SEM and a two-sample equal variance t-test was used. *p<0.05, **p<0.01 compared to control group (Ctrl).
(EPS)

Figure S2 **RT-PCR confirmation of concordant epigenes in spinal cord.** Total RNA was extracted from postmortem human spinal cord tissue used for the methylation analysis from sALS subjects (n = 8–11) and controls (n = 8–11) and subjected to RT-PCR. Results were normalized to housekeeping genes [TATA-box Binding Protein (*TBP*) for *CTSZ*, *FCER1G, TREM2*, *NRN1* and *NNAT*; ribosomal 18S subunit for *CHI3L2*, *H19*, *PEG10*, and *LUM*], and are presented as fold-changes calculated by the 2^−ΔΔC^_T_ method. *p<0.05, **p<0.01, ***p<0.001, ****p<0.0001.
(EPS)

Table S1 Samples used for methylation and expression analyses.
(DOC)

Table S2 Detailed comparison of concordant and common epigenes to other high-throughput data sets.
(DOC)

Table S3 Human oligonucleotide sequences of primers used for pyrosequencing.
(DOC)

Table S4 Human oligonucleotide sequences of primers used for real-time RT-PCR.
(DOC)
