# Supplementary material for: Identification of Epigenetically Altered Genes in Sporadic Amyotrophic Lateral Sclerosis
Source: PLoS One. 2012 Dec 26;7(12):e52672. doi: 10.1371/journal.pone.0052672 (PMC3530456; doi:10.1371/journal.pone.0052672)
Supplement: Table S1 — Samples used for methylation and expression analyses. (DOC) [file pone.0052672.s003.doc]

**Table S1. Samples used for methylation and expression analyses.**

|  | **sALS postmortem spinal cord samples** | | | | | | **Control postmortem spinal cord samples** | | | | | | **Whole blood samples** | | | | | |
| --- | --- | --- | --- | --- | --- | --- | --- | --- | --- | --- | --- | --- | --- | --- | --- | --- | --- | --- |
|  | **NICHD code** | **5mC Array** | **Gene Expression Array** | **RT-PCR** | **G5mC** | **G5HmC** | **NICHD code** | **5mC Array** | **Gene Expression Array** | **RT-PCR** | **G5mC** | **G5HmC** | **MICHR code** | **sALS**  **G5mC** | **sALS G5hmC** | **MICHR Code** | **CtrlG5mC** | **Ctrl G5hmC** |
| 1 | UMB_5015 | Y | O | Y | O | NT | UMB_0772 | Y | Y | Y | Y | Y | ALS0013 | Y | Y | ALS0042C | Y | Y |
| 2 | UMB_4627 | Y | Y | Y | Y | Y | UMB_4781 | Y | Y | Y | Y | Y | ALS0034 | Y | Y | ALS0158C | Y | Y |
| 3 | UMB_1292 | Y | Y | Y | Y | Y | UMB_1228 | Y | Y | Y | Y | Y | ALS0295 | Y | Y | ALS0168C | Y | Y |
| 4 | UMB_4768 | Y | Y | Y | Y | Y | UMB_0145 | Y | Y | Y | Y | Y | ALS0311 | Y | O | ALS0195C | Y | O |
| 5 | UMB_0925 | Y | Y | Y | Y | Y | UMB_1131 | Y | Y | Y | Y | Y | ALS0353 | Y | Y | ALS0241C | Y | Y |
| 6 | M3580M | Y | O | Y | Y | Y | UMB_1471 | Y | Y | Y | Y | Y | ALS0396 | Y | Y | ALS0354C | Y | Y |
| 7 | UMB_4762 | Y | Y | Y | y | Y | UMB_0840 | Y | O | Y | Y | Y | ALS0552 | Y | Y | ALS0355C | Y | Y |
| 8 | UMB_1314 | Y | Y | Y | Y | Y | UMB_4263 | Y | O | Y | Y | Y | ALS0719 | Y | Y | ALS0611C | Y | Y |
| 9 | M3226M | Y | Y | Y | Y | Y | UMB_4735 | Y | Y | Y | O | Y | ALS0931 | Y | Y | ALS0686C | Y | O |
| 10 | UMB_1081 | Y | Y | Y | Y | Y | UMB_4257 | Y | Y | Y | Y | Y | ALS1105 | Y | Y | ALS0746C | Y | Y |
| 11 | UMB_1100 | Y | Y | Y | Y | Y | M4022M | Y | NH | Y | Y | Y | ALS1148 | Y | O | ALS1021C | NT | Y |
| 12 | UMB_0357 | NT | Y | NT | NT | NT |  |  |  |  | NT | NT |  |  |  | ALS1059C | Y | O |

NICHD , the National Center for Child Health and Human Development; Y, sample used for hybridization and/or analysis; O, outlier samples not used in the analysis; NH, not hybridized (low RNA quality); NT, not tested; 5mC, 5-methylCytosine; 5HmC, 5-HydroxyMethylCytosine; G5mC, global 5-methylCytosine; G5HmC, global 5-HydroxyMethylCytosine.; Ctrl, control.
