# Supplementary material for: Identification of Epigenetically Altered Genes in Sporadic Amyotrophic Lateral Sclerosis
Source: PLoS One. 2012 Dec 26;7(12):e52672. doi: 10.1371/journal.pone.0052672 (PMC3530456; doi:10.1371/journal.pone.0052672)
Supplement: Table S2 — Detailed comparison of concordant and common epigenes to other high-throughput data sets. (DOC) [file pone.0052672.s004.doc]

# Table S2. Detailed comparison of concordant and common epigenes to other high-throughput data sets

| **cpgSiteID** | **EntrezID** | **Symbol** | **Description** | Set Count | DMG | DEG | COMMON | CONCORDANT | ALSoD | Malsapina2001 | Dangold2004 | Jiang2005MN | Jiang2005VH | Wang2006 | Lederer2007 | Morahan2009 | Kirby2011 |
| --- | --- | --- | --- | --- | --- | --- | --- | --- | --- | --- | --- | --- | --- | --- | --- | --- | --- |
| cg01309152 | 5121 | PCP4 | Purkinje cell protein 4 | 7 | 15.9 | -2.0 | ✓ | ✓ | ✓ | • | • | • | • | • | 0.5 | Hyper | • |
| cg10634424 | 4060 | LUM | lumican | 5 | 37.0 | -4.1 | ✓ | ✓ | ✓ | • | • | • | • | • | • | • | • |
| cg10503138 | 152330 | CNTN4 | contactin 4 | 5 | 48.8 | -1.6 | ✓ | ✓ | ✓ | • | • | • | • | • | • | • | • |
| cg05485060 | 8727 | CTNNAL1 | catenin (cadherin-associated protein), alpha-like 1 | 6 | 17.1 | -2.5 | ✓ | ✓ | • | • | • | • | • | -2.4 | • | • | -2.3 |
| cg23208881 | 5045 | FURIN | furin (paired basic amino acid cleaving enzyme) | 6 | -19.7 | 1.6 | ✓ | ✓ | • | • | • | -3.3 | -2.2 | • | • | • | • |
| cg15699524 | 8817 | FGF18 | fibroblast growth factor 18 | 6 | 15.8 | -1.4 | ✓ | ✓ | • | • | • | -2.6 | • | • | • | • | -3.1 |
| cg17399166 | 912 | CD1D | CD1d molecule | 5 | -19.7 | 2.8 | ✓ | ✓ | • | • | • | • | -2.2 | • | • | • | • |
| cg04151683 | 158038 | LINGO2 | leucine rich repeat and Ig domain containing 2 | 5 | 42.7 | -3.3 | ✓ | ✓ | • | • | • | • | • | • | • | • | 3.6 |
| cg15514848 | 2326 | FMO1 | flavin containing monooxygenase 1 | 5 | 61.1 | -1.5 | ✓ | ✓ | • | -5.0 | • | • | • | • | • | • | • |
| cg05675373 | 3749 | KCNC4 | potassium voltage-gated channel, Shaw-related subfamily, member 4 | 5 | 28.2 | -1.3 | ✓ | ✓ | • | • | • | • | • | • | • | • | 2.8 |
| cg20334738 | 10586 | MAB21L2 | mab-21-like 2 (C. elegans) | 5 | 19.5 | -1.9 | ✓ | ✓ | • | • | • | • | • | • | • | • | 2.7 |
| cg06197492 | 283120 | H19 | H19, imprinted maternally expressed transcript (non-protein coding) | 4 | 37.1 | -2.3 | ✓ | ✓ | • | • | • | • | • | • | • | • | • |
| cg02879662 | 64344 | HIF3A | hypoxia inducible factor 3, alpha subunit | 4 | -48.9 | 2.1 | ✓ | ✓ | • | • | • | • | • | • | • | • | • |
| cg20609368 | 3045 | HBD | hemoglobin, delta | 4 | 77.9 | -2.3 | ✓ | ✓ | • | • | • | • | • | • | • | • | • |
| cg25918245 | 26157 | GIMAP2 | GTPase, IMAP family member 2 | 4 | -23.4 | 2.2 | ✓ | ✓ | • | • | • | • | • | • | • | • | • |
| cg06836736 | 4199 | ME1 | malic enzyme 1, NADP(+)-dependent, cytosolic | 4 | 15.2 | -1.7 | ✓ | ✓ | • | • | • | • | • | • | • | • | • |
| cg14767877 | 5660 | PSAP | prosaposin | 4 | -33.5 | 1.3 | ✓ | ✓ | • | • | • | • | • | • | • | • | • |
| cg27446233 | 5213 | PFKM | phosphofructokinase, muscle | 4 | 29.3 | -1.4 | ✓ | ✓ | • | • | • | • | • | • | • | • | • |
| cg00629217 | 84708 | LNX1 | ligand of numb-protein X 1 | 4 | 30.5 | -1.9 | ✓ | ✓ | • | • | • | • | • | • | • | • | • |
| cg14646244 | 5172 | SLC26A4 | solute carrier family 26, member 4 | 4 | 23.5 | -1.5 | ✓ | ✓ | • | • | • | • | • | • | • | • | • |
| cg08927738 | 8537 | BCAS1 | breast carcinoma amplified sequence 1 | 4 | 59.2 | -1.4 | ✓ | ✓ | • | • | • | • | • | • | • | • | • |
| cg22647507 | 23089 | PEG10 | paternally expressed 10 | 4 | 13.2 | -1.8 | ✓ | ✓ | • | • | • | • | • | • | • | • | • |
| cg08722122 | 2260 | FGFR1 | fibroblast growth factor receptor 1 | 4 | 18.7 | -1.3 | ✓ | ✓ | • | • | • | • | • | • | • | • | • |
| cg13022129 | 254295 | PHYHD1 | phytanoyl-CoA dioxygenase domain containing 1 | 4 | -13.6 | 1.9 | ✓ | ✓ | • | • | • | • | • | • | • | • | • |
| cg06790862 | 744 | MPPED2 | metallophosphoesterase domain containing 2 | 4 | 17.4 | -1.3 | ✓ | ✓ | • | • | • | • | • | • | • | • | • |
| cg22642718 | 3768 | KCNJ12 | potassium inwardly-rectifying channel, subfamily J, member 12 | 4 | 343.9 | -1.4 | ✓ | ✓ | • | • | • | • | • | • | • | • | • |
| cg19191401 | 57522 | SRGAP1 | SLIT-ROBO Rho GTPase activating protein 1 | 4 | -43.9 | 1.5 | ✓ | ✓ | • | • | • | • | • | • | • | • | • |
| cg24881834 | 4199 | ME1 | malic enzyme 1, NADP(+)-dependent, cytosolic | 4 | 18.6 | -1.7 | ✓ | ✓ | • | • | • | • | • | • | • | • | • |
| cg00186701 | 85453 | TSPYL5 | TSPY-like 5 | 4 | 32.0 | -1.4 | ✓ | ✓ | • | • | • | • | • | • | • | • | • |
| cg13281868 | 90523 | C6orf142 | muscular LMNA-interacting protein | 4 | 343.9 | -4.1 | ✓ | ✓ | • | • | • | • | • | • | • | • | • |
| cg22419732 | 254228 | FAM26E | family with sequence similarity 26, member E | 4 | 97.0 | -1.8 | ✓ | ✓ | • | • | • | • | • | • | • | • | • |
| cg12563178 | 84898 | PLXDC2 | plexin domain containing 2 | 4 | -23.7 | 2.0 | ✓ | ✓ | • | • | • | • | • | • | • | • | • |
| cg24736099 | 5332 | PLCB4 | phospholipase C, beta 4 | 4 | 49.5 | -1.8 | ✓ | ✓ | • | • | • | • | • | • | • | • | • |
| cg17296166 | 55228 | PNMAL1 | PNMA-like 1 | 4 | 30.6 | -1.6 | ✓ | ✓ | • | • | • | • | • | • | • | • | • |
| cg23134169 | 79939 | SLC35E1 | solute carrier family 35, member E1 | 4 | -46.6 | 1.3 | ✓ | ✓ | • | • | • | • | • | • | • | • | • |
| cg05922591 | 11006 | LILRB4 | leukocyte immunoglobulin-like receptor, subfamily B (with TM and ITIM domains), member 4 | 4 | -14.0 | 2.7 | ✓ | ✓ | • | • | • | • | • | • | • | • | • |
| cg01663968 | 1522 | CTSZ | cathepsin Z | 4 | -61.8 | 2.2 | ✓ | ✓ | • | • | • | • | • | • | • | • | • |
| cg23458892 | 27036 | SIGLEC7 | sialic acid binding Ig-like lectin 7 | 4 | -15.9 | 2.7 | ✓ | ✓ | • | • | • | • | • | • | • | • | • |
| cg17491456 | 1601 | DAB2 | disabled homolog 2, mitogen-responsive phosphoprotein (Drosophila) | 4 | -13.7 | 2.0 | ✓ | ✓ | • | • | • | • | • | • | • | • | • |
| cg18960218 | 9056 | SLC7A7 | solute carrier family 7 (amino acid transporter light chain, y+L system), member 7 | 4 | -18.6 | 3.5 | ✓ | ✓ | • | • | • | • | • | • | • | • | • |
| cg03001305 | 6776 | STAT5A | signal transducer and activator of transcription 5A | 4 | -16.6 | 1.6 | ✓ | ✓ | • | • | • | • | • | • | • | • | • |
| cg11979312 | 8743 | TNFSF10 | tumor necrosis factor (ligand) superfamily, member 10 | 4 | 33.0 | -2.2 | ✓ | ✓ | • | • | • | • | • | • | • | • | • |
| cg23566503 | 4826 | NNAT | neuronatin | 4 | 15.3 | -2.2 | ✓ | ✓ | • | • | • | • | • | • | • | • | • |
| cg17274742 | 10457 | GPNMB | glycoprotein (transmembrane) nmb | 4 | -43.1 | 2.8 | ✓ | ✓ | • | • | • | • | • | • | • | • | • |
| cg10853416 | 58475 | MS4A7 | membrane-spanning 4-domains, subfamily A, member 7 | 4 | -19.0 | 4.6 | ✓ | ✓ | • | • | • | • | • | • | • | • | • |
| cg07440414 | 6932 | TCF7 | transcription factor 7 (T-cell specific, HMG-box) | 4 | 87.4 | -1.7 | ✓ | ✓ | • | • | • | • | • | • | • | • | • |
| cg04881903 | 822 | CAPG | capping protein (actin filament), gelsolin-like | 4 | -13.9 | 3.4 | ✓ | ✓ | • | • | • | • | • | • | • | • | • |
| cg26158194 | 4046 | LSP1 | lymphocyte-specific protein 1 | 4 | -20.1 | 2.7 | ✓ | ✓ | • | • | • | • | • | • | • | • | • |
| cg11976790 | 1755 | DMBT1 | deleted in malignant brain tumors 1 | 4 | 24.1 | -2.3 | ✓ | ✓ | • | • | • | • | • | • | • | • | • |
| cg25302419 | 1501 | CTNND2 | catenin (cadherin-associated protein), delta 2 (neural plakophilin-related arm-repeat protein) | 4 | -18.0 | 1.6 | ✓ | ✓ | • | • | • | • | • | • | • | • | • |
| cg19690404 | 51200 | CPA4 | carboxypeptidase A4 | 4 | 66.0 | -2.4 | ✓ | ✓ | • | • | • | • | • | • | • | • | • |
| cg25463779 | 144347 | FAM101A | family with sequence similarity 101, member A | 4 | 33.9 | -1.8 | ✓ | ✓ | • | • | • | • | • | • | • | • | • |
| cg00321478 | 23418 | CRB1 | crumbs homolog 1 (Drosophila) | 4 | -15.2 | 1.7 | ✓ | ✓ | • | • | • | • | • | • | • | • | • |
| cg00412772 | 64073 | C19orf33 | chromosome 19 open reading frame 33 | 4 | 29.2 | -3.4 | ✓ | ✓ | • | • | • | • | • | • | • | • | • |
| cg08684473 | 10990 | LILRB5 | leukocyte immunoglobulin-like receptor, subfamily B (with TM and ITIM domains), member 5 | 4 | -36.6 | 2.0 | ✓ | ✓ | • | • | • | • | • | • | • | • | • |
| cg20654468 | 9404 | LPXN | leupaxin | 4 | -13.9 | 1.5 | ✓ | ✓ | • | • | • | • | • | • | • | • | • |
| cg19389852 | 10628 | TXNIP | thioredoxin interacting protein | 4 | -26.1 | 1.4 | ✓ | ✓ | • | • | • | • | • | • | • | • | • |
| cg20178764 | 2676 | GFRA3 | GDNF family receptor alpha 3 | 4 | 19.3 | -2.0 | ✓ | ✓ | • | • | • | • | • | • | • | • | • |
| cg05659526 | 2207 | FCER1G | Fc fragment of IgE, high affinity I, receptor for; gamma polypeptide | 4 | -20.2 | 2.4 | ✓ | ✓ | • | • | • | • | • | • | • | • | • |
| cg26808784 | 283120 | H19 | H19, imprinted maternally expressed transcript (non-protein coding) | 4 | 70.5 | -2.3 | ✓ | ✓ | • | • | • | • | • | • | • | • | • |
| cg01227741 | 168537 | GIMAP7 | GTPase, IMAP family member 7 | 4 | 31.1 | -1.7 | ✓ | ✓ | • | • | • | • | • | • | • | • | • |
| cg16179125 | 1522 | CTSZ | cathepsin Z | 4 | -40.8 | 2.2 | ✓ | ✓ | • | • | • | • | • | • | • | • | • |
| cg25989745 | 7306 | TYRP1 | tyrosinase-related protein 1 | 4 | 50.0 | -7.2 | ✓ | ✓ | • | • | • | • | • | • | • | • | • |
| cg08077345 | 57664 | PLEKHA4 | pleckstrin homology domain containing, family A (phosphoinositide binding specific) member 4 | 4 | 26.4 | -2.4 | ✓ | ✓ | • | • | • | • | • | • | • | • | • |
| cg21750589 | 64288 | ZNF323 | zinc finger protein 323 | 4 | 37.9 | -1.9 | ✓ | ✓ | • | • | • | • | • | • | • | • | • |
| cg27394566 | 122618 | PLD4 | phospholipase D family, member 4 | 4 | -18.0 | 1.5 | ✓ | ✓ | • | • | • | • | • | • | • | • | • |
| cg24919884 | 27237 | ARHGEF16 | Rho guanine nucleotide exchange factor (GEF) 16 | 4 | 101.6 | -1.5 | ✓ | ✓ | • | • | • | • | • | • | • | • | • |
| cg25852472 | 283120 | H19 | H19, imprinted maternally expressed transcript (non-protein coding) | 4 | 37.2 | -2.3 | ✓ | ✓ | • | • | • | • | • | • | • | • | • |
| cg23577242 | 744 | MPPED2 | metallophosphoesterase domain containing 2 | 4 | 66.3 | -1.3 | ✓ | ✓ | • | • | • | • | • | • | • | • | • |
| cg25511429 | 51299 | NRN1 | neuritin 1 | 4 | 18.1 | -2.5 | ✓ | ✓ | • | • | • | • | • | • | • | • | • |
| cg17371067 | 1051 | CEBPB | CCAAT/enhancer binding protein (C/EBP), beta | 4 | -54.7 | 2.1 | ✓ | ✓ | • | • | • | • | • | • | • | • | • |
| cg26453670 | 55544 | RBM38 | RNA binding motif protein 38 | 4 | -20.8 | 1.7 | ✓ | ✓ | • | • | • | • | • | • | • | • | • |
| cg27015047 | 1755 | DMBT1 | deleted in malignant brain tumors 1 | 4 | 48.6 | -2.3 | ✓ | ✓ | • | • | • | • | • | • | • | • | • |
| cg02005755 | 26266 | SLC13A4 | solute carrier family 13 (sodium/sulfate symporters), member 4 | 4 | 26.2 | -4.2 | ✓ | ✓ | • | • | • | • | • | • | • | • | • |
| cg02082571 | 50856 | CLEC4A | C-type lectin domain family 4, member A | 4 | -45.3 | 1.7 | ✓ | ✓ | • | • | • | • | • | • | • | • | • |
| cg25299676 | 4233 | MET | met proto-oncogene (hepatocyte growth factor receptor) | 4 | 22.9 | -1.4 | ✓ | ✓ | • | • | • | • | • | • | • | • | • |
| cg15652212 | 135932 | TMEM139 | transmembrane protein 139 | 4 | 26.9 | -1.7 | ✓ | ✓ | • | • | • | • | • | • | • | • | • |
| cg13144783 | 1230 | CCR1 | chemokine (C-C motif) receptor 1 | 4 | -25.2 | 2.3 | ✓ | ✓ | • | • | • | • | • | • | • | • | • |
| cg13564468 | 116843 | C6orf192 | chromosome 6 open reading frame 192 | 4 | -16.5 | 1.7 | ✓ | ✓ | • | • | • | • | • | • | • | • | • |
| cg10863038 | 64859 | OBFC2A | oligonucleotide/oligosaccharide-binding fold containing 2A | 4 | -99.8 | 1.5 | ✓ | ✓ | • | • | • | • | • | • | • | • | • |
| cg18950617 | 23526 | HMHA1 | histocompatibility (minor) HA-1 | 4 | -33.5 | 1.8 | ✓ | ✓ | • | • | • | • | • | • | • | • | • |
| cg13021192 | 1522 | CTSZ | cathepsin Z | 4 | -15.4 | 2.2 | ✓ | ✓ | • | • | • | • | • | • | • | • | • |
| cg17169998 | 23209 | MLC1 | megalencephalic leukoencephalopathy with subcortical cysts 1 | 4 | -16.6 | 1.7 | ✓ | ✓ | • | • | • | • | • | • | • | • | • |
| cg16335762 | 123920 | CMTM3 | CKLF-like MARVEL transmembrane domain containing 3 | 4 | -16.6 | 1.6 | ✓ | ✓ | • | • | • | • | • | • | • | • | • |
| cg10642330 | 4826 | NNAT | neuronatin | 4 | 26.3 | -2.2 | ✓ | ✓ | • | • | • | • | • | • | • | • | • |
| cg18433380 | 4826 | NNAT | neuronatin | 4 | 21.6 | -2.2 | ✓ | ✓ | • | • | • | • | • | • | • | • | • |
| cg27096144 | 4488 | MSX2 | msh homeobox 2 | 4 | -52.0 | 1.9 | ✓ | ✓ | • | • | • | • | • | • | • | • | • |
| cg04757093 | 864 | RUNX3 | runt-related transcription factor 3 | 4 | -32.8 | 2.6 | ✓ | ✓ | • | • | • | • | • | • | • | • | • |
| cg19534945 | 286256 | LCN12 | lipocalin 12 | 4 | -18.5 | 2.3 | ✓ | ✓ | • | • | • | • | • | • | • | • | • |
| cg15432938 | 10023 | FRAT1 | frequently rearranged in advanced T-cell lymphomas | 4 | -46.0 | 2.2 | ✓ | ✓ | • | • | • | • | • | • | • | • | • |
| cg19320612 | 6335 | SCN9A | sodium channel, voltage-gated, type IX, alpha subunit | 4 | 47.3 | -1.8 | ✓ | ✓ | • | • | • | • | • | • | • | • | • |
| cg11054936 | 2706 | GJB2 | gap junction protein, beta 2, 26kDa | 4 | 18.4 | -4.7 | ✓ | ✓ | • | • | • | • | • | • | • | • | • |
| cg19979896 | 135250 | RAET1E | retinoic acid early transcript 1E | 4 | 89.7 | -1.3 | ✓ | ✓ | • | • | • | • | • | • | • | • | • |
| cg21533958 | 25960 | GPR124 | G protein-coupled receptor 124 | 4 | 18.2 | -1.8 | ✓ | ✓ | • | • | • | • | • | • | • | • | • |
| cg15865742 | 1317 | SLC31A1 | solute carrier family 31 (copper transporters), member 1 | 4 | -19.4 | 1.6 | ✓ | ✓ | • | • | • | • | • | • | • | • | • |
| cg21405195 | 23089 | PEG10 | paternally expressed 10 | 4 | 20.6 | -1.8 | ✓ | ✓ | • | • | • | • | • | • | • | • | • |
| cg20095587 | 54209 | TREM2 | triggering receptor expressed on myeloid cells 2 | 4 | -26.0 | 2.9 | ✓ | ✓ | • | • | • | • | • | • | • | • | • |
| cg23486067 | 5359 | PLSCR1 | phospholipid scramblase 1 | 4 | -82.6 | 1.8 | ✓ | ✓ | • | • | • | • | • | • | • | • | • |
| cg09473585 | 57415 | C3orf14 | chromosome 3 open reading frame 14 | 4 | 78.2 | -1.5 | ✓ | ✓ | • | • | • | • | • | • | • | • | • |
| cg21400896 | 51225 | ABI3 | ABI family, member 3 | 4 | -19.2 | 2.3 | ✓ | ✓ | • | • | • | • | • | • | • | • | • |
| cg26277709 | 84909 | C9orf3 | chromosome 9 open reading frame 3 | 4 | 40.4 | -1.5 | ✓ | ✓ | • | • | • | • | • | • | • | • | • |
| cg06791867 | 90139 | TSPAN18 | tetraspanin 18 | 4 | 24.8 | -2.0 | ✓ | ✓ | • | • | • | • | • | • | • | • | • |
| cg13214422 | 91977 | MYOZ3 | myozenin 3 | 4 | 15.3 | -1.3 | ✓ | ✓ | • | • | • | • | • | • | • | • | • |
| cg22456522 | 11025 | LILRB3 | leukocyte immunoglobulin-like receptor, subfamily B (with TM and ITIM domains), member 3 | 4 | -14.0 | 2.5 | ✓ | ✓ | • | • | • | • | • | • | • | • | • |
| cg11172423 | 149461 | CLDN19 | claudin 19 | 4 | 31.6 | -4.1 | ✓ | ✓ | • | • | • | • | • | • | • | • | • |
| cg05628549 | 112464 | PRKCDBP | protein kinase C, delta binding protein | 4 | 28.6 | -3.8 | ✓ | ✓ | • | • | • | • | • | • | • | • | • |
| cg01488147 | 23089 | PEG10 | paternally expressed 10 | 4 | 29.7 | -1.8 | ✓ | ✓ | • | • | • | • | • | • | • | • | • |
| cg16356956 | 1439 | CSF2RB | colony stimulating factor 2 receptor, beta, low-affinity (granulocyte-macrophage) | 4 | -35.4 | 3.8 | ✓ | ✓ | • | • | • | • | • | • | • | • | • |
| cg00625425 | 163782 | KANK4 | KN motif and ankyrin repeat domains 4 | 4 | 100.3 | -2.4 | ✓ | ✓ | • | • | • | • | • | • | • | • | • |
| cg10045881 | 1117 | CHI3L2 | chitinase 3-like 2 | 4 | -22.7 | 2.1 | ✓ | ✓ | • | • | • | • | • | • | • | • | • |
| cg06590610 | 10320 | IKZF1 | IKAROS family zinc finger 1 (Ikaros) | 4 | -22.2 | 2.1 | ✓ | ✓ | • | • | • | • | • | • | • | • | • |
| cg00044729 | 150350 | ENTHD1 | ENTH domain containing 1 | 4 | 81.9 | -1.5 | ✓ | ✓ | • | • | • | • | • | • | • | • | • |
| cg01714932 | 5858 | PZP | pregnancy-zone protein | 4 | 48.7 | -2.0 | ✓ | ✓ | • | • | • | • | • | • | • | • | • |
| cg20850981 | 64288 | ZNF323 | zinc finger protein 323 | 4 | 27.4 | -1.9 | ✓ | ✓ | • | • | • | • | • | • | • | • | • |
| cg20663831 | 26157 | GIMAP2 | GTPase, IMAP family member 2 | 4 | -46.7 | 2.2 | ✓ | ✓ | • | • | • | • | • | • | • | • | • |
| cg10564498 | 558 | AXL | AXL receptor tyrosine kinase | 4 | -14.5 | 1.8 | ✓ | ✓ | • | • | • | • | • | • | • | • | • |
| cg26759551 | 2203 | FBP1 | fructose-1,6-bisphosphatase 1 | 4 | -96.5 | 2.7 | ✓ | ✓ | • | • | • | • | • | • | • | • | • |
| cg05861567 | 23209 | MLC1 | megalencephalic leukoencephalopathy with subcortical cysts 1 | 4 | -41.3 | 1.7 | ✓ | ✓ | • | • | • | • | • | • | • | • | • |
| cg13044136 | 166 | AES | amino-terminal enhancer of split | 4 | 42.7 | -1.4 | ✓ | ✓ | • | • | • | • | • | • | • | • | • |
| cg22843446 | 79827 | ASAM | CXADR-like membrane protein | 4 | 87.6 | -1.6 | ✓ | ✓ | • | • | • | • | • | • | • | • | • |
| cg02497428 | 10261 | IGSF6 | immunoglobulin superfamily, member 6 | 4 | -50.2 | 2.3 | ✓ | ✓ | • | • | • | • | • | • | • | • | • |
| cg00845900 | 51200 | CPA4 | carboxypeptidase A4 | 4 | 28.7 | -2.4 | ✓ | ✓ | • | • | • | • | • | • | • | • | • |
| cg09450238 | 90135 | BTBD6 | BTB (POZ) domain containing 6 | 4 | 23.6 | -1.3 | ✓ | ✓ | • | • | • | • | • | • | • | • | • |
| cg21283680 | 9467 | SH3BP5 | SH3-domain binding protein 5 (BTK-associated) | 4 | 50.6 | -1.5 | ✓ | ✓ | • | • | • | • | • | • | • | • | • |
| cg15739944 | 22927 | HABP4 | hyaluronan binding protein 4 | 4 | 45.1 | -1.4 | ✓ | ✓ | • | • | • | • | • | • | • | • | • |
| cg18140857 | 195814 | SDR16C5 | short chain dehydrogenase/reductase family 16C, member 5 | 4 | 16.7 | -3.5 | ✓ | ✓ | • | • | • | • | • | • | • | • | • |
| cg18854666 | 6556 | SLC11A1 | solute carrier family 11 (proton-coupled divalent metal ion transporters), member 1 | 4 | -34.4 | 3.1 | ✓ | ✓ | • | • | • | • | • | • | • | • | • |
| cg24597988 | 6402 | SELL | selectin L | 4 | 82.5 | 1.6 | ✓ | • | ✓ | • | • | • | • | • | • | • | • |
| cg13765621 | 912 | CD1D | CD1d molecule | 5 | 14.9 | 2.8 | ✓ | • | • | • | • | • | -2.2 | • | • | • | • |
| cg08705994 | 864 | RUNX3 | runt-related transcription factor 3 | 4 | 29.6 | 2.6 | ✓ | • | • | • | • | • | • | • | • | • | • |
| cg20586531 | 5359 | PLSCR1 | phospholipid scramblase 1 | 4 | 89.4 | 1.8 | ✓ | • | • | • | • | • | • | • | • | • | • |
| cg19107595 | 23089 | PEG10 | paternally expressed 10 | 4 | -20.3 | -1.8 | ✓ | • | • | • | • | • | • | • | • | • | • |
| cg07022477 | 64344 | HIF3A | hypoxia inducible factor 3, alpha subunit | 4 | 16.2 | 2.1 | ✓ | • | • | • | • | • | • | • | • | • | • |
| cg00113020 | 11006 | LILRB4 | leukocyte immunoglobulin-like receptor, subfamily B (with TM and ITIM domains), member 4 | 4 | 29.1 | 2.7 | ✓ | • | • | • | • | • | • | • | • | • | • |
| cg11504897 | 51299 | NRN1 | neuritin 1 | 4 | -16.7 | -2.5 | ✓ | • | • | • | • | • | • | • | • | • | • |
| cg18813928 | 864 | RUNX3 | runt-related transcription factor 3 | 4 | 20.7 | 2.6 | ✓ | • | • | • | • | • | • | • | • | • | • |
| cg23919867 | 864 | RUNX3 | runt-related transcription factor 3 | 4 | 22.7 | 2.6 | ✓ | • | • | • | • | • | • | • | • | • | • |
| cg06037693 | 864 | RUNX3 | runt-related transcription factor 3 | 4 | 29.2 | 2.6 | ✓ | • | • | • | • | • | • | • | • | • | • |
| cg18117847 | 10320 | IKZF1 | IKAROS family zinc finger 1 (Ikaros) | 4 | 37.5 | 2.1 | ✓ | • | • | • | • | • | • | • | • | • | • |
| cg26377677 | 5045 | FURIN | furin (paired basic amino acid cleaving enzyme) | 6 | 60.3 | 1.6 | ✓ | • | • | • | • | -3.3 | -2.2 | • | • | • | • |
| cg24612198 | 916 | CD3E | CD3e molecule, epsilon (CD3-TCR complex) | 5 | 23.8 | 1.8 | ✓ | • | • | • | • | -5.6 | -2.2 | • | • | • | • |
| cg22373112 | 5878 | RAB5C | RAB5C, member RAS oncogene family | 5 | 14.6 | 1.4 | ✓ | • | • | • | • | -2.5 | -3.2 | • | • | • | • |
| cg14301635 | 717 | C2 | complement component 2 | 5 | 17.9 | 2.0 | ✓ | • | • | • | • | 2.9 | 3.6 | • | • | • | • |
| cg16097772 | 4069 | LYZ | lysozyme | 5 | 14.8 | 2.9 | ✓ | • | • | • | • | 2.0 | 3.9 | • | • | • | • |
| cg11916609 | 9173 | IL1RL1 | interleukin 1 receptor-like 1 | 4 | 34.6 | 2.1 | ✓ | • | • | • | • | • | • | • | • | • | 5.2 |
| cg26537639 | 1535 | CYBA | cytochrome b-245, alpha polypeptide | 4 | 25.5 | 2.2 | ✓ | • | • | • | 10.3 | • | • | • | • | • | • |
| cg05064181 | 3983 | ABLIM1 | actin binding LIM protein 1 | 4 | 50.2 | 1.4 | ✓ | • | • | • | • | -5.3 | • | • | • | • | • |
| cg19790294 | 1535 | CYBA | cytochrome b-245, alpha polypeptide | 4 | 36.4 | 2.2 | ✓ | • | • | • | 10.3 | • | • | • | • | • | • |
| cg16386158 | 9173 | IL1RL1 | interleukin 1 receptor-like 1 | 4 | 49.9 | 2.1 | ✓ | • | • | • | • | • | • | • | • | • | 5.2 |
| cg17834443 | 55174 | INTS10 | integrator complex subunit 10 | 4 | 36.0 | 1.4 | ✓ | • | • | • | • | • | • | • | • | • | -2.8 |
| cg10970409 | 7433 | VIPR1 | vasoactive intestinal peptide receptor 1 | 4 | 16.3 | 1.7 | ✓ | • | • | • | • | -4.4 | • | • | • | • | • |
| cg23665603 | 23541 | SEC14L2 | SEC14-like 2 (S. cerevisiae) | 4 | 15.6 | 1.8 | ✓ | • | • | • | • | • | • | • | • | • | 2.3 |
| cg08347960 | 6326 | SCN2A | sodium channel, voltage-gated, type II, alpha subunit | 4 | -14.6 | -1.8 | ✓ | • | • | • | • | • | • | • | • | • | 2.9 |
| cg22975568 | 972 | CD74 | CD74 molecule, major histocompatibility complex, class II invariant chain | 4 | 19.5 | 2.4 | ✓ | • | • | • | 4.4 | • | • | • | • | • | • |
| cg09294589 | 10491 | CRTAP | cartilage associated protein | 4 | 18.5 | 1.3 | ✓ | • | • | • | • | • | • | • | • | • | -3.0 |
| cg23620639 | 1305 | COL13A1 | collagen, type XIII, alpha 1 | 4 | -65.9 | -1.4 | ✓ | • | • | • | • | • | • | • | • | • | -7.9 |
| cg20932053 | 1368 | CPM | carboxypeptidase M | 4 | 67.5 | 2.6 | ✓ | • | • | • | • | • | • | • | • | • | 2.3 |
| cg01683883 | 146225 | CMTM2 | CKLF-like MARVEL transmembrane domain containing 2 | 4 | 25.2 | 1.4 | ✓ | • | • | • | • | • | • | • | • | • | -3.5 |
| cg00051623 | 834 | CASP1 | caspase 1, apoptosis-related cysteine peptidase (interleukin 1, beta, convertase) | 4 | 60.3 | 2.0 | ✓ | • | • | • | • | 2.2 | • | • | • | • | • |
| cg26594488 | 54103 | PION | pigeon homolog (Drosophila) | 4 | 61.3 | 1.8 | ✓ | • | • | • | • | • | • | -2.4 | • | • | • |
| cg11577097 | 366 | AQP9 | aquaporin 9 | 3 | 18.9 | 3.3 | ✓ | • | • | • | • | • | • | • | • | • | • |
| cg04450876 | 121355 | GTSF1 | gametocyte specific factor 1 | 3 | 30.6 | 2.0 | ✓ | • | • | • | • | • | • | • | • | • | • |
| cg23983366 | 26953 | RANBP6 | RAN binding protein 6 | 3 | -14.4 | -1.4 | ✓ | • | • | • | • | • | • | • | • | • | • |
| cg15185001 | 51599 | LSR | lipolysis stimulated lipoprotein receptor | 3 | -64.5 | -1.5 | ✓ | • | • | • | • | • | • | • | • | • | • |
| cg04799664 | 84166 | NLRC5 | NLR family, CARD domain containing 5 | 3 | 39.1 | 1.6 | ✓ | • | • | • | • | • | • | • | • | • | • |
| cg10210238 | 1030 | CDKN2B | cyclin-dependent kinase inhibitor 2B (p15, inhibits CDK4) | 3 | 35.4 | 1.4 | ✓ | • | • | • | • | • | • | • | • | • | • |
| cg00141162 | 3059 | HCLS1 | hematopoietic cell-specific Lyn substrate 1 | 3 | 21.3 | 3.0 | ✓ | • | • | • | • | • | • | • | • | • | • |
| cg04681849 | 5157 | PDGFRL | platelet-derived growth factor receptor-like | 3 | -23.5 | -2.0 | ✓ | • | • | • | • | • | • | • | • | • | • |
| cg14116596 | 6916 | TBXAS1 | thromboxane A synthase 1 (platelet) | 3 | 30.6 | 3.2 | ✓ | • | • | • | • | • | • | • | • | • | • |
| cg03845435 | 128346 | C1orf162 | chromosome 1 open reading frame 162 | 3 | 21.6 | 2.7 | ✓ | • | • | • | • | • | • | • | • | • | • |
| cg25189085 | 246329 | STAC3 | SH3 and cysteine rich domain 3 | 3 | 28.1 | 1.5 | ✓ | • | • | • | • | • | • | • | • | • | • |
| cg19481686 | 1030 | CDKN2B | cyclin-dependent kinase inhibitor 2B (p15, inhibits CDK4) | 3 | 343.9 | 1.4 | ✓ | • | • | • | • | • | • | • | • | • | • |
| cg14150666 | 3579 | IL8RB | chemokine (C-X-C motif) receptor 2 | 3 | 20.7 | 2.1 | ✓ | • | • | • | • | • | • | • | • | • | • |
| cg23752985 | 8673 | VAMP8 | vesicle-associated membrane protein 8 (endobrevin) | 3 | 30.3 | 2.1 | ✓ | • | • | • | • | • | • | • | • | • | • |
| cg26861460 | 64098 | PARVG | parvin, gamma | 3 | 25.8 | 2.0 | ✓ | • | • | • | • | • | • | • | • | • | • |
| cg13059335 | 27299 | ADAMDEC1 | ADAM-like, decysin 1 | 3 | 76.0 | 3.9 | ✓ | • | • | • | • | • | • | • | • | • | • |
| cg16282679 | 80864 | EGFL8 | EGF-like-domain, multiple 8 | 3 | -35.9 | -2.1 | ✓ | • | • | • | • | • | • | • | • | • | • |
| cg04675937 | 1030 | CDKN2B | cyclin-dependent kinase inhibitor 2B (p15, inhibits CDK4) | 3 | 85.6 | 1.4 | ✓ | • | • | • | • | • | • | • | • | • | • |
| cg19464016 | 639 | PRDM1 | PR domain containing 1, with ZNF domain | 3 | 17.1 | 2.4 | ✓ | • | • | • | • | • | • | • | • | • | • |
| cg12365667 | 3115 | HLA-DPB1 | major histocompatibility complex, class II, DP beta 1 | 3 | 16.8 | 1.8 | ✓ | • | • | • | • | • | • | • | • | • | • |
| cg19157971 | 5877 | RABIF | RAB interacting factor | 3 | -26.6 | -1.4 | ✓ | • | • | • | • | • | • | • | • | • | • |
| cg11797883 | 81873 | ARPC5L | actin related protein 2/3 complex, subunit 5-like | 3 | -23.2 | -1.4 | ✓ | • | • | • | • | • | • | • | • | • | • |
| cg06827976 | 2268 | FGR | Gardner-Rasheed feline sarcoma viral (v-fgr) oncogene homolog | 3 | 18.8 | 2.8 | ✓ | • | • | • | • | • | • | • | • | • | • |
| cg08700651 | 163702 | IL28RA | interleukin 28 receptor, alpha (interferon, lambda receptor) | 3 | 16.7 | 1.8 | ✓ | • | • | • | • | • | • | • | • | • | • |
| cg14740251 | 8778 | SIGLEC5 | sialic acid binding Ig-like lectin 5 | 3 | 41.7 | 2.1 | ✓ | • | • | • | • | • | • | • | • | • | • |
| cg16470760 | 920 | CD4 | CD4 molecule | 3 | 19.5 | 2.2 | ✓ | • | • | • | • | • | • | • | • | • | • |
| cg17927917 | 3638 | INSIG1 | insulin induced gene 1 | 3 | -29.5 | -1.5 | ✓ | • | • | • | • | • | • | • | • | • | • |
| cg05365670 | 79888 | LPCAT1 | lysophosphatidylcholine acyltransferase 1 | 3 | 21.5 | 1.4 | ✓ | • | • | • | • | • | • | • | • | • | • |
| cg16685860 | 5338 | PLD2 | phospholipase D2 | 3 | 35.3 | 1.5 | ✓ | • | • | • | • | • | • | • | • | • | • |
| cg15364618 | 27141 | CIDEB | cell death-inducing DFFA-like effector b | 3 | 28.9 | 1.7 | ✓ | • | • | • | • | • | • | • | • | • | • |
| cg01668126 | 4481 | MSR1 | macrophage scavenger receptor 1 | 3 | 16.0 | 1.4 | ✓ | • | • | • | • | • | • | • | • | • | • |
| cg00394658 | 5795 | PTPRJ | protein tyrosine phosphatase, receptor type, J | 3 | 45.4 | 1.5 | ✓ | • | • | • | • | • | • | • | • | • | • |
| cg14831798 | 9425 | CDYL | chromodomain protein, Y-like | 3 | 77.3 | 1.3 | ✓ | • | • | • | • | • | • | • | • | • | • |
| cg26718420 | 120939 | C12orf59 | chromosome 12 open reading frame 59 | 3 | 65.2 | 2.1 | ✓ | • | • | • | • | • | • | • | • | • | • |
| cg24924631 | 597 | BCL2A1 | BCL2-related protein A1 | 3 | 32.1 | 2.1 | ✓ | • | • | • | • | • | • | • | • | • | • |
| cg09600520 | 9425 | CDYL | chromodomain protein, Y-like | 3 | 32.0 | 1.3 | ✓ | • | • | • | • | • | • | • | • | • | • |
| cg27104271 | 11309 | SLCO2B1 | solute carrier organic anion transporter family, member 2B1 | 3 | 27.7 | 1.9 | ✓ | • | • | • | • | • | • | • | • | • | • |
| cg16584172 | 7546 | ZIC2 | Zic family member 2 | 3 | -37.4 | -2.2 | ✓ | • | • | • | • | • | • | • | • | • | • |
| cg07063745 | 10314 | LANCL1 | LanC lantibiotic synthetase component C-like 1 (bacterial) | 3 | -29.2 | -1.4 | ✓ | • | • | • | • | • | • | • | • | • | • |
| cg23818978 | 120425 | AMICA1 | adhesion molecule, interacts with CXADR antigen 1 | 3 | 29.0 | 2.0 | ✓ | • | • | • | • | • | • | • | • | • | • |
| cg05755354 | 55691 | FRMD4A | FERM domain containing 4A | 3 | 13.6 | 1.4 | ✓ | • | • | • | • | • | • | • | • | • | • |
| cg21992250 | 51296 | SLC15A3 | solute carrier family 15, member 3 | 3 | 32.0 | 2.7 | ✓ | • | • | • | • | • | • | • | • | • | • |
| cg14433673 | 7351 | UCP2 | uncoupling protein 2 (mitochondrial, proton carrier) | 3 | 18.3 | 1.9 | ✓ | • | • | • | • | • | • | • | • | • | • |
| cg12207371 | 9332 | CD163 | CD163 molecule | 3 | 30.0 | 3.2 | ✓ | • | • | • | • | • | • | • | • | • | • |
| cg19252956 | 81704 | DOCK8 | dedicator of cytokinesis 8 | 3 | 46.0 | 1.8 | ✓ | • | • | • | • | • | • | • | • | • | • |
| cg23960723 | 79746 | ECHDC3 | enoyl CoA hydratase domain containing 3 | 3 | 17.9 | 2.3 | ✓ | • | • | • | • | • | • | • | • | • | • |
| cg18117393 | 80896 | NPL | N-acetylneuraminate pyruvate lyase (dihydrodipicolinate synthase) | 3 | 48.4 | 2.9 | ✓ | • | • | • | • | • | • | • | • | • | • |
| cg17631429 | 2307 | FOXS1 | forkhead box S1 | 3 | -15.5 | -2.3 | ✓ | • | • | • | • | • | • | • | • | • | • |
| cg25017250 | 346 | APOC4 | apolipoprotein C-IV | 3 | 21.2 | 1.6 | ✓ | • | • | • | • | • | • | • | • | • | • |
| cg22189286 | 26353 | HSPB8 | heat shock 22kDa protein 8 | 3 | 16.1 | 1.8 | ✓ | • | • | • | • | • | • | • | • | • | • |
| cg24456340 | 2793 | GNGT2 | guanine nucleotide binding protein (G protein), gamma transducing activity polypeptide 2 | 3 | 37.6 | 2.0 | ✓ | • | • | • | • | • | • | • | • | • | • |
| cg18979223 | 1030 | CDKN2B | cyclin-dependent kinase inhibitor 2B (p15, inhibits CDK4) | 3 | 24.7 | 1.4 | ✓ | • | • | • | • | • | • | • | • | • | • |
| cg15032239 | 23191 | CYFIP1 | cytoplasmic FMR1 interacting protein 1 | 3 | 16.9 | 1.3 | ✓ | • | • | • | • | • | • | • | • | • | • |
| cg22143352 | 122953 | JDP2 | Jun dimerization protein 2 | 3 | 36.5 | 1.6 | ✓ | • | • | • | • | • | • | • | • | • | • |
| cg21540749 | 4338 | MOCS2 | molybdenum cofactor synthesis 2 | 3 | -33.0 | -1.3 | ✓ | • | • | • | • | • | • | • | • | • | • |
| cg22295573 | 361 | AQP4 | aquaporin 4 | 3 | 14.3 | 1.7 | ✓ | • | • | • | • | • | • | • | • | • | • |
| cg18988395 | 169792 | GLIS3 | GLIS family zinc finger 3 | 3 | 19.0 | 1.9 | ✓ | • | • | • | • | • | • | • | • | • | • |
| cg00098162 | 118932 | ANKRD22 | ankyrin repeat domain 22 | 3 | 31.9 | 3.0 | ✓ | • | • | • | • | • | • | • | • | • | • |
| cg01889448 | 3119 | HLA-DQB1 | major histocompatibility complex, class II, DQ beta 1 | 3 | 13.9 | 2.6 | ✓ | • | • | • | • | • | • | • | • | • | • |
| cg06812844 | 7226 | TRPM2 | transient receptor potential cation channel, subfamily M, member 2 | 3 | 17.3 | 1.6 | ✓ | • | • | • | • | • | • | • | • | • | • |
| cg15727320 | 8909 | P11 | endonuclease, polyU-specific | 3 | 43.3 | 1.7 | ✓ | • | • | • | • | • | • | • | • | • | • |
| cg25697050 | 3635 | INPP5D | inositol polyphosphate-5-phosphatase, 145kDa | 3 | 32.5 | 1.5 | ✓ | • | • | • | • | • | • | • | • | • | • |
| cg27143049 | 5140 | PDE3B | phosphodiesterase 3B, cGMP-inhibited | 3 | 16.4 | 2.0 | ✓ | • | • | • | • | • | • | • | • | • | • |
| cg14904464 | 4753 | NELL2 | NEL-like 2 (chicken) | 3 | -56.8 | -2.0 | ✓ | • | • | • | • | • | • | • | • | • | • |
| cg04705866 | 3003 | GZMK | granzyme K (granzyme 3; tryptase II) | 3 | 28.9 | 3.0 | ✓ | • | • | • | • | • | • | • | • | • | • |
| cg06147863 | 6688 | SPI1 | spleen focus forming virus (SFFV) proviral integration oncogene spi1 | 3 | 17.2 | 1.9 | ✓ | • | • | • | • | • | • | • | • | • | • |
| cg07264679 | 9332 | CD163 | CD163 molecule | 3 | 20.0 | 3.2 | ✓ | • | • | • | • | • | • | • | • | • | • |
| cg11256445 | 3059 | HCLS1 | hematopoietic cell-specific Lyn substrate 1 | 3 | 46.0 | 3.0 | ✓ | • | • | • | • | • | • | • | • | • | • |
| cg14154330 | 201176 | ARHGAP27 | Rho GTPase activating protein 27 | 3 | 38.0 | 1.4 | ✓ | • | • | • | • | • | • | • | • | • | • |
| cg14088811 | 6688 | SPI1 | spleen focus forming virus (SFFV) proviral integration oncogene spi1 | 3 | 30.6 | 1.9 | ✓ | • | • | • | • | • | • | • | • | • | • |
| cg07596401 | 64077 | LHPP | phospholysine phosphohistidine inorganic pyrophosphate phosphatase | 3 | -13.1 | -1.6 | ✓ | • | • | • | • | • | • | • | • | • | • |
| cg06720660 | 6039 | RNASE6 | ribonuclease, RNase A family, k6 | 3 | 26.1 | 3.2 | ✓ | • | • | • | • | • | • | • | • | • | • |
| cg00221494 | 10160 | FARP1 | FERM, RhoGEF (ARHGEF) and pleckstrin domain protein 1 (chondrocyte-derived) | 3 | 21.2 | 1.6 | ✓ | • | • | • | • | • | • | • | • | • | • |
| cg00548060 | 80896 | NPL | N-acetylneuraminate pyruvate lyase (dihydrodipicolinate synthase) | 3 | 29.0 | 2.9 | ✓ | • | • | • | • | • | • | • | • | • | • |
| cg13912117 | 114 | ADCY8 | adenylate cyclase 8 (brain) | 3 | 53.0 | 1.9 | ✓ | • | • | • | • | • | • | • | • | • | • |
| cg09516965 | 5729 | PTGDR | prostaglandin D2 receptor (DP) | 3 | -24.7 | -2.4 | ✓ | • | • | • | • | • | • | • | • | • | • |
| cg17869167 | 8809 | IL18R1 | interleukin 18 receptor 1 | 3 | 60.7 | 2.2 | ✓ | • | • | • | • | • | • | • | • | • | • |
| cg13760253 | 85479 | DNAJC5B | DnaJ (Hsp40) homolog, subfamily C, member 5 beta | 3 | 43.1 | 4.7 | ✓ | • | • | • | • | • | • | • | • | • | • |
| cg08719486 | 1612 | DAPK1 | death-associated protein kinase 1 | 3 | 19.0 | 1.8 | ✓ | • | • | • | • | • | • | • | • | • | • |
| cg06148264 | 1992 | SERPINB1 | serpin peptidase inhibitor, clade B (ovalbumin), member 1 | 3 | 56.2 | 1.6 | ✓ | • | • | • | • | • | • | • | • | • | • |
| cg01579216 | 4688 | NCF2 | neutrophil cytosolic factor 2 | 3 | 53.4 | 3.0 | ✓ | • | • | • | • | • | • | • | • | • | • |
| cg18084791 | 56833 | SLAMF8 | SLAM family member 8 | 3 | 46.2 | 5.3 | ✓ | • | • | • | • | • | • | • | • | • | • |
| cg16753209 | 4703 | NEB | nebulin | 3 | 56.2 | 1.7 | ✓ | • | • | • | • | • | • | • | • | • | • |
| cg08390209 | 1030 | CDKN2B | cyclin-dependent kinase inhibitor 2B (p15, inhibits CDK4) | 3 | 33.2 | 1.4 | ✓ | • | • | • | • | • | • | • | • | • | • |
| cg07259382 | 55016 | MARCH1 | membrane-associated ring finger (C3HC4) 1 | 3 | 71.1 | 1.4 | ✓ | • | • | • | • | • | • | • | • | • | • |
| cg14754581 | 9034 | CCRL2 | chemokine (C-C motif) receptor-like 2 | 3 | 27.6 | 2.9 | ✓ | • | • | • | • | • | • | • | • | • | • |
| cg02945019 | 8832 | CD84 | CD84 molecule | 3 | 14.8 | 1.9 | ✓ | • | • | • | • | • | • | • | • | • | • |
| cg21130374 | 4600 | MX2 | myxovirus (influenza virus) resistance 2 (mouse) | 3 | 30.9 | 2.3 | ✓ | • | • | • | • | • | • | • | • | • | • |
| cg25464840 | 55691 | FRMD4A | FERM domain containing 4A | 3 | 16.6 | 1.4 | ✓ | • | • | • | • | • | • | • | • | • | • |
| cg03404502 | 2841 | GPR18 | G protein-coupled receptor 18 | 3 | 72.9 | 1.6 | ✓ | • | • | • | • | • | • | • | • | • | • |
| cg01697865 | 3587 | IL10RA | interleukin 10 receptor, alpha | 3 | 49.5 | 4.2 | ✓ | • | • | • | • | • | • | • | • | • | • |
| cg26980692 | 51296 | SLC15A3 | solute carrier family 15, member 3 | 3 | 15.9 | 2.7 | ✓ | • | • | • | • | • | • | • | • | • | • |
| cg16719404 | 914 | CD2 | CD2 molecule | 3 | 17.1 | 2.2 | ✓ | • | • | • | • | • | • | • | • | • | • |
| cg26099316 | 3699 | ITIH3 | inter-alpha-trypsin inhibitor heavy chain 3 | 3 | -14.8 | -3.9 | ✓ | • | • | • | • | • | • | • | • | • | • |
| cg21171615 | 5788 | PTPRC | protein tyrosine phosphatase, receptor type, C | 3 | 72.5 | 2.2 | ✓ | • | • | • | • | • | • | • | • | • | • |
| cg14143055 | 27299 | ADAMDEC1 | ADAM-like, decysin 1 | 3 | 72.6 | 3.9 | ✓ | • | • | • | • | • | • | • | • | • | • |
| cg14386061 | 9046 | DOK2 | docking protein 2, 56kDa | 3 | 25.3 | 1.4 | ✓ | • | • | • | • | • | • | • | • | • | • |
| cg01795122 | 2014 | EMP3 | epithelial membrane protein 3 | 3 | 15.4 | 1.6 | ✓ | • | • | • | • | • | • | • | • | • | • |
| cg05303448 | 65985 | AACS | acetoacetyl-CoA synthetase | 3 | -25.0 | -1.4 | ✓ | • | • | • | • | • | • | • | • | • | • |
| cg02807450 | 8898 | MTMR2 | myotubularin related protein 2 | 3 | -13.6 | -1.3 | ✓ | • | • | • | • | • | • | • | • | • | • |
| cg15875120 | 80013 | C10orf97 | family with sequence similarity 188, member A | 3 | -36.2 | -1.5 | ✓ | • | • | • | • | • | • | • | • | • | • |
| cg10979891 | 64093 | SMOC1 | SPARC related modular calcium binding 1 | 3 | 16.3 | 1.8 | ✓ | • | • | • | • | • | • | • | • | • | • |
| cg00186954 | 6764 | ST5 | suppression of tumorigenicity 5 | 3 | 21.1 | 1.5 | ✓ | • | • | • | • | • | • | • | • | • | • |
| cg17751569 | 4064 | CD180 | CD180 molecule | 3 | 42.9 | 2.6 | ✓ | • | • | • | • | • | • | • | • | • | • |
| cg19279346 | 10288 | LILRB2 | leukocyte immunoglobulin-like receptor, subfamily B (with TM and ITIM domains), member 2 | 3 | 41.0 | 2.5 | ✓ | • | • | • | • | • | • | • | • | • | • |
| cg07086380 | 25816 | TNFAIP8 | tumor necrosis factor, alpha-induced protein 8 | 3 | 27.0 | 1.7 | ✓ | • | • | • | • | • | • | • | • | • | • |
| cg03430998 | 7096 | TLR1 | toll-like receptor 1 | 3 | 25.1 | 1.9 | ✓ | • | • | • | • | • | • | • | • | • | • |
| cg08023751 | 10461 | MERTK | c-mer proto-oncogene tyrosine kinase | 3 | 58.5 | 2.7 | ✓ | • | • | • | • | • | • | • | • | • | • |
| cg14642338 | 25891 | PAMR1 | peptidase domain containing associated with muscle regeneration 1 | 3 | 18.4 | 1.9 | ✓ | • | • | • | • | • | • | • | • | • | • |
| cg02813863 | 81575 | APOLD1 | apolipoprotein L domain containing 1 | 3 | 22.0 | 1.8 | ✓ | • | • | • | • | • | • | • | • | • | • |
| cg19556572 | 80709 | AKNA | AT-hook transcription factor | 3 | 76.6 | 2.2 | ✓ | • | • | • | • | • | • | • | • | • | • |
| cg12343777 | 247 | ALOX15B | arachidonate 15-lipoxygenase, type B | 3 | 22.1 | 5.1 | ✓ | • | • | • | • | • | • | • | • | • | • |
| cg27623214 | 147685 | C19orf18 | chromosome 19 open reading frame 18 | 3 | 24.0 | 1.8 | ✓ | • | • | • | • | • | • | • | • | • | • |
| cg19635810 | 54978 | C2orf18 | chromosome 2 open reading frame 18 | 3 | 47.3 | 1.3 | ✓ | • | • | • | • | • | • | • | • | • | • |
| cg09646392 | 10673 | TNFSF13B | tumor necrosis factor (ligand) superfamily, member 13b | 3 | 54.1 | 1.9 | ✓ | • | • | • | • | • | • | • | • | • | • |
| cg07685869 | 3801 | KIFC3 | kinesin family member C3 | 3 | -21.1 | -1.5 | ✓ | • | • | • | • | • | • | • | • | • | • |
| cg08663969 | 9619 | ABCG1 | ATP-binding cassette, sub-family G (WHITE), member 1 | 3 | 125.0 | 2.3 | ✓ | • | • | • | • | • | • | • | • | • | • |
| cg26244225 | 81575 | APOLD1 | apolipoprotein L domain containing 1 | 3 | 110.0 | 1.8 | ✓ | • | • | • | • | • | • | • | • | • | • |
| cg01773854 | 3489 | IGFBP6 | insulin-like growth factor binding protein 6 | 3 | -29.5 | -2.5 | ✓ | • | • | • | • | • | • | • | • | • | • |
| cg15799267 | 247 | ALOX15B | arachidonate 15-lipoxygenase, type B | 3 | 13.6 | 5.1 | ✓ | • | • | • | • | • | • | • | • | • | • |
| cg11154542 | 1514 | CTSL1 | cathepsin L1 | 3 | 16.0 | 1.4 | ✓ | • | • | • | • | • | • | • | • | • | • |
| cg17090012 | 1612 | DAPK1 | death-associated protein kinase 1 | 3 | 55.3 | 1.8 | ✓ | • | • | • | • | • | • | • | • | • | • |
| cg17836145 | 8875 | VNN2 | vanin 2 | 3 | 23.7 | 2.7 | ✓ | • | • | • | • | • | • | • | • | • | • |
| cg03359508 | 311 | ANXA11 | annexin A11 | 3 | 22.8 | 1.7 | ✓ | • | • | • | • | • | • | • | • | • | • |
| cg24248317 | 54978 | C2orf18 | chromosome 2 open reading frame 18 | 3 | 14.0 | 1.3 | ✓ | • | • | • | • | • | • | • | • | • | • |
| cg09584711 | 3250 | HPR | haptoglobin-related protein | 3 | 58.9 | 2.1 | ✓ | • | • | • | • | • | • | • | • | • | • |
| cg17439694 | 1356 | CP | ceruloplasmin (ferroxidase) | 3 | 16.3 | 1.9 | ✓ | • | • | • | • | • | • | • | • | • | • |
| cg23776012 | 125113 | KRT222 | keratin 222 | 3 | -34.1 | -5.4 | ✓ | • | • | • | • | • | • | • | • | • | • |
| cg10925082 | 397 | ARHGDIB | Rho GDP dissociation inhibitor (GDI) beta | 3 | 24.2 | 1.8 | ✓ | • | • | • | • | • | • | • | • | • | • |
| cg13119609 | 344 | APOC2 | apolipoprotein C-II | 3 | 13.9 | 2.2 | ✓ | • | • | • | • | • | • | • | • | • | • |
| cg01805540 | 783 | CACNB2 | calcium channel, voltage-dependent, beta 2 subunit | 3 | -40.0 | -1.4 | ✓ | • | • | • | • | • | • | • | • | • | • |
| cg19826026 | 397 | ARHGDIB | Rho GDP dissociation inhibitor (GDI) beta | 3 | 17.1 | 1.8 | ✓ | • | • | • | • | • | • | • | • | • | • |
| cg10150530 | 4033 | LRMP | lymphoid-restricted membrane protein | 3 | 14.9 | 2.2 | ✓ | • | • | • | • | • | • | • | • | • | • |
| cg07026910 | 3635 | INPP5D | inositol polyphosphate-5-phosphatase, 145kDa | 3 | 18.1 | 1.5 | ✓ | • | • | • | • | • | • | • | • | • | • |
| cg16301617 | 11322 | TMC6 | transmembrane channel-like 6 | 3 | 22.9 | 1.4 | ✓ | • | • | • | • | • | • | • | • | • | • |
| cg20320468 | 3903 | LAIR1 | leukocyte-associated immunoglobulin-like receptor 1 | 3 | 65.1 | 2.2 | ✓ | • | • | • | • | • | • | • | • | • | • |
| cg14847483 | 51234 | TMEM85 | transmembrane protein 85 | 3 | -23.2 | -1.3 | ✓ | • | • | • | • | • | • | • | • | • | • |
| cg17271365 | 55016 | MARCH1 | membrane-associated ring finger (C3HC4) 1 | 3 | 30.9 | 1.4 | ✓ | • | • | • | • | • | • | • | • | • | • |
| cg07689731 | 9672 | SDC3 | syndecan 3 | 3 | 49.6 | 1.5 | ✓ | • | • | • | • | • | • | • | • | • | • |

This table summarizes the overlapping among the concordant (and common) epigenes from the current study, previously published microarray studies, and the genes in the ALSoD database (<http://alsod.iop.kcl.ac.uk/>). ‘Set count’ column refers to the total number of data sets (out of the 13) including the gene. The first four data columns (DMG, DEG, COMMON, and CONCORDANT) are from the current study. The columns in blue (DMGs from our study and Morahan et al. ) are from methylation profiling assays and the values in DMG column correspond to the DiffScore of the differential methylation (See Method for more details). Numeric values in other data columns are gene expression fold-changes (positive values: up-regulation; negative values: down-regulation in ALS samples). ‘Hyper’ and ‘Hypo’ stand for hyper-methylated and hypo-methylated in the ALS samples, respectively. ‘✓’ in the data columns indicate that the corresponding gene was identified as differentially methylated/expressed (or both). ‘•’ in the data columns indicate that the corresponding gene was not differentially methylated/expressed (or both).

# References for Table S2.
