# Supplementary material for: Identification of Epigenetically Altered Genes in Sporadic Amyotrophic Lateral Sclerosis
Source: PLoS One. 2012 Dec 26;7(12):e52672. doi: 10.1371/journal.pone.0052672 (PMC3530456; doi:10.1371/journal.pone.0052672)
Supplement: Table S3 — Human oligonucleotide sequences of primers used for pyrosequencing. (DOC) [file pone.0052672.s005.doc]

**Table S3. Human oligonucleotide sequences of primers used for pyrosequencing**

| **Gene** | **Primers** | **Primer Sequence** | **Annealing Temperature** |
| --- | --- | --- | --- |
| **CMTM2** |  |  |  |
| Forward | CMTM2F | 5'-AAGTTGAAAGGAAGTTTTTTTT-3' | 47 °C |
| Reverse (Biotinylated | CMTM2R* | 5'-BIOTIN-GTTTAAATGTGATTATAAAATTAG-3' |  |
| Sequencing Forward | CMTM2F/SEQ | 5'-AACCCCAAATCTATAAACC-3' |  |
| Nucleotide dispensation |  | 5'-CCCGGTTGTTGTTTTTAT**Y**GAGAATATTTGTTTTTTTT**Y**GTTGTTTTGTGTGAAAGT**Y**G**Y**  GTTGTATTTTTTTG**Y**GTTTGG**Y**GTTG**Y**GTT**Y**G**Y**GG-3' |  |
| **CMTM3** |  |  |  |
| Forward | CMTM3F/SEQ | 5'-TAGGAAGTTGGGTTTTGATTA-3' | 55 °C |
| Reverse (Biotinylated | CMTM3R* | 5'-BIOTIN-CAACTCCAAACACACACACAA-3' |  |
| Sequencing Forward | CMTM3F/SEQ | 5'-TAGGAAGTTGGGTTTTGATTA-3' |  |
| Nucleotide dispensation |  | **5**'**-Y**GTTGAATTTTAGA**Y**GTTTTTTAGATGTGTA**YG**TATGTG**Y**GTGTGGAGTGTGTGTGTGT  GTGTGTGTGA-3' |  |
| **CXCL12** |  |  |  |
| Forward (Biotinylated | CXCL12For* | 5'-BIOTIN-GTTTTTTGTTTTGTTTGTA-3' | 40 °C |
| Reverse | CXCL12Rev | 5'-AACAAACAAATTAATC-3' |  |
| Sequencing Forward 1 | CXCL12RevSeq | 5'-AAAATCCTACTTTCTAT--3' |  |
| Sequencing Forward 2 | CXCL12For* | 5'-BIOTIN-GTTTTTTGTTTTGTTTGTA-3' |  |
| Nucleotide dispensation |  | 5'-CCCAC**R**TAAAAACTCAAACTC**R**CCACCTACCC**R**ACTTAC**R**AC**R**AC**R**CAACC**R**CC**R**ACAA  AACTC**R**ATCTAC**R**AAAATAAAACCC**R**TCTT-3' |  |
|  |  | 5'-CCCCC**R**AAC**R**AAAAACCTACTAAC**R**ATAAC**R**ACCCAAAACAACC**R**AAAAAATCCTACTT  TCTATAC**R**TAAAAACTCAAACTC**R**CCA-3' |  |
| **STAT5A** |  |  |  |
| Forward | STAT5AFor1 | 5'-GAGGAGAGGGAAGTTGGGTAAA-3' | 57 °C |
| Reverse (Biotinylated) | STAT5ARev1* | 5'-BIOTIN-CCACCCAAAACCACACAATAA-3' |  |
| Sequencing Forward | STAT5ASeq1F | 5'-AGGGAAGTTGGGTAAAG-3' |  |
|  | STAT5ASeq2F | 5'-TGGTAAGGTTTGTAGAGAGT-3' |  |
|  | STAT5ASeq3F | 5'-AGTTTTATAAGTAATTAGGTTGGGT-3' |  |
| Nucleotide dispensation |  | 5'-GGGATGGAAGGYGTTTAGTTYGATTTTATTAAATTTTTTGGGTTTYGTGGGAAGGGGTT |  |
|  |  | TTTTGGAGAGGGGGATTGAGGTTTTAGATAGGATATTTATTGTTGTGGTAAGGTTTGTA |  |
|  |  | GAGAGTTTYGAAGTTAGGAGGATTTAAGAYGGTTTTTTTTTGGATTTTTTTGAAGGTAG  AATTAGTTTTATAAGTAATTAGGTTGGGTGAAYGGGGGYGTTGGTTAGTTTATGGATTA  TAGTYGGTTGGTGAGGTTAYGTGTTTATTGTGTGGTTTTGGGTGGTTT-3' |  |
| **C/EBPB** |  |  |  |
| Forward | C/EBPBFor | 5'-GGGTTTAGGAGAAATTTTAG-3' | 50 °C |
| Reverse (Biotinylated) | C/EBPBRev2* | 5'-BIOTIN-ACCTCCCTACTCTAAACTATC-3' |  |
| Sequencing Forward | C/EBPBSeq5 | 5'-GGGTTTAGGAGAAATTTT-3' |  |
| Nucleotide dispensation |  | 5'-AG**Y**GAGTTAGAGT**Y**G**Y**GTA**Y**GGGATTGGGAAGGGGATTTAT-3' |  |
|  |  | 5'-GGGATGGAAGG**Y**GTTTAGTT**Y**GATTTTATTAAATTTTTTGGGTTT**Y**GTGGGAAGGGGTT  TTTTGGAGAGGGGGATTGAGGTTTTAGATAGGATATTTATTGTTG-3' |  |
|  |  | 5'-GAAGTTAGGAGGATTTAAGA**Y**GGTTTTTTTTTGGATTTTTTTGAAGGTAGAATT-3' |  |
|  |  | 5'-GGGGG**Y**GTTGGTTAGTTTATGGATTATAGT**Y**GGTTGGTGAGGTTA**Y**GTGTTTATTGTGT  GGTTTTGGGTGGTTT-3' |  |
