# Supplementary material for: Identification of Epigenetically Altered Genes in Sporadic Amyotrophic Lateral Sclerosis
Source: PLoS One. 2012 Dec 26;7(12):e52672. doi: 10.1371/journal.pone.0052672 (PMC3530456; doi:10.1371/journal.pone.0052672)
Supplement: Table S4 — Human oligonucleotide sequences of primers used for real-time RT-PCR. (DOC) [file pone.0052672.s006.doc]

**Table S4. Human oligonucleotide sequences of primers used for real-time RT-PCR**

|  |  | **Primers** | **Primer Sequence** | **Annealing Temperature (^o^C)** | **Reference** |
| --- | --- | --- | --- | --- | --- |
| *CTSZ* | 1522 | hCTSZF | 5'-GAGTGTGACAAGTTTAACCAATGTG-3' | 56 |  |
|  |  | hCTSZR | 5'-GGTGTAGTTAGCCAGTCTTTCTGTT-3' |  |  |
| *CHI3L2* | 1117 | hCHI3L2F | 5'-CCATGGACCAGAAGTCTCTC-3' | 55 |  |
|  |  | hCHI3L2R | 5'-ATGCTGGCGAATGAATAGAT-3' |  |  |
| *FURIN* | 5045 | hFurinF | 5'-GTACACACAGATGAATGACAACAGG-3' | 58 |  |
|  |  | hFurinR | 5'-CACTGTAGATGTGGATGTGGTTG-3' |  |  |
| *FMO1* | 2326 | hFMO1F | 5'-GCTTAATACCAGAAGACAGGACTCA-3' | 58 |  |
|  |  | hFMO1R | 5'-AGACAATGATGTCAATAGGCTCTTC-3' | |  |
| *GJB2* | 2706 | hGJB2F | 5'-AAGAACGTGTGCTACGATCACTACT-3' | 58 |  |
|  |  | hGJB2R | 5'-TGAACTTCCTCTTCTTCTCATGTCT-3' |  |  |
| *FCER1G* | 2207 | hFCER1GF | 5'-ATGATTCCAGCAGTGGTCTTG-3' | 56 |  |
|  |  | hFCER1GR | 5'-CTTTCGCACTTGGATCTTCAG-3' |  |  |
| *H19* | 283120 | H19-10248 | 5'-CTTTACAACCACTGCACTACCTGAC-3' | 60 |  |
|  |  | H19-10423 | 5'-GATGGTGTCTTTGATGTTGGGCTGA-3' | |  |
| *LUM* | 4060 | hLUMF | 5'-CTCTCTTCCTGGCATTGATT-3' | 55 |  |
|  |  | hLUMR | 5'-AGGCACCATTGGTACACTTT-3' |  |  |
| *PEG10* | 23089 | ABP-F2 | 5'-CAGAGGAGTCCTCGCGTG-3' | 60 |  |
|  |  | ABP-R2 | 5'-GGATGGAGGCCTGGATCC-3' |  |  |
| *NRN1* | 51299 | NRN1-2F | 5'-CGAAAGATATGTGGGATAAACTGAG-3' | 58 |  |
|  |  | NRN1-2R | 5'-CTAAAGCTGCCGAGAGAGACAC-3' |  |  |
| *NNAT* | 4826 | hNNAT-F | 5'-TGCTGCATTTACTGGGTAGG-3' | 58 |  |
|  |  | hNNAT-R | 5'-ACACCGTGTATGCCAGCTTC-3' |  |  |
| *SLC11A1* | 6556 | hSLC11A1F | 5′-AGCGGACATCAGAGAAGCCAACAT-3′ | 60 |  |
|  |  | hSLC11A1R | 5′-CTGCCCAAAGACAGCCATGACAAA-3′ | |  |
| STAT5A | 6776 | hSTAT5AF | 5'–GTTCAGTGTTGGCAGCAATGAGC–3' | 58 |  |
|  |  | hSTAT5AR | 5'–AGCACAGTAGCCGTGGCATTGT–3' |  |  |
| *TREM2* | 54209 | hTREM2F | 5'-GCATCTTTCTCATCAAGATTCTAGC-3' | 56 |  |
|  |  | hTREM2R | 5'-CTGGCAGAGTTTGGAGCTGATAC-3' |  |  |
| *18S* | 100008588 | h18SF | 5'-TTCGGAACTGAGGC ATGAT-3' | 55-60 |  |
|  |  | h18SR | 5'-TTTCGCTCTGGTCCGTCTTG-3' |  |  |
| *TBP* | 6908 | hTBPF | 5'-GAGCCAGAGTTATTTCCTGGTTTA-3' | 55-60 |  |
|  |  | hTBPF | 5'-ATTTCTGCTCTGACTTTAGCACCT-3' |  |  |
| *GAPDH* | 2597 | hGAPDG-F | 5'-GTGGTCTCCTCTGACTTCAACAG-3' | 55-60 |  |
|  |  | hGAPDH-R | 5'-CTGTAGCCAAATTCGTTGTCATAC-3' |  |  |

# References for Table S4.
